# Biallelic *ADAM22* pathogenic variants cause progressive encephalopathy and infantile-onset refractory epilepsy

**DOI:** 10.1093/brain/awac116

**Published:** 2022-04-04

**Authors:** Marieke M van der Knoop, Reza Maroofian, Yuko Fukata, Yvette van Ierland, Ehsan G Karimiani, Anna Elina Lehesjoki, Mikko Muona, Anders Paetau, Yuri Miyazaki, Yoko Hirano, Laila Selim, Marina de França, Rodrigo Ambrosio Fock, Christian Beetz, Claudia A L Ruivenkamp, Alison J Eaton, Francois D Morneau-Jacob, Lena Sagi-Dain, Lilach Shemer-Meiri, Amir Peleg, Jumana Haddad-Halloun, Daan J Kamphuis, Cacha M P C D Peeters-Scholte, Semra Hiz Kurul, Rita Horvath, Hanns Lochmüller, David Murphy, Stephan Waldmüller, Stephanie Spranger, David Overberg, Alison M Muir, Aboulfazl Rad, Barbara Vona, Firdous Abdulwahad, Sateesh Maddirevula, Inna S Povolotskaya, Victoria Y Voinova, Vykuntaraju K Gowda, Varunvenkat M Srinivasan, Fowzan S Alkuraya, Heather C Mefford, Majid Alfadhel, Tobias B Haack, Pasquale Striano, Mariasavina Severino, Masaki Fukata, Yvonne Hilhorst-Hofstee, Henry Houlden

**Affiliations:** Department of Child Neurology, Sophia Children’s Hospital, Erasmus University Medical Center, 3015 CN Rotterdam, The Netherlands; Department of Neuromuscular Disorders, UCL Queen Square Institute of Neurology, University College London, London WC1N 3BG, UK; Division of Membrane Physiology, Department of Molecular and Cellular Physiology, National Institute for Physiological Sciences, National Institutes of Natural Sciences, Okazaki, Aichi 444-8787, Japan; Department of Physiological Sciences, School of Life Science, SOKENDAI (The Graduate University for Advanced Studies), Okazaki, Aichi 444-8585, Japan; Department of Clinical Genetics, Erasmus University Medical Center, 3015 CN Rotterdam, The Netherlands; Next Generation Genetic Polyclinic, Razavi International Hospital, Mashhad, Iran; Genetics Research Centre, Molecular and Clinical Sciences Institute, St. George’s University, London SW17 0RE, UK; Folkhälsan Research Center, Department of Medical and Clinical Genetics, Medicum, University of Helsinki, Helsinki 00290, Finland; Folkhälsan Research Center, Department of Medical and Clinical Genetics, Medicum, University of Helsinki, Helsinki 00290, Finland; Department of Medical and Clinical Genetics, Medicum, University of Helsinki, Finland, 00100 Helsinki, Finland; Blueprint Genetics, 02150 Espoo, Finland; Department of Pathology, Medicum, University of Helsinki, 00100 Helsinki, Finland; Division of Membrane Physiology, Department of Molecular and Cellular Physiology, National Institute for Physiological Sciences, National Institutes of Natural Sciences, Okazaki, Aichi 444-8787, Japan; Department of Physiological Sciences, School of Life Science, SOKENDAI (The Graduate University for Advanced Studies), Okazaki, Aichi 444-8585, Japan; Division of Membrane Physiology, Department of Molecular and Cellular Physiology, National Institute for Physiological Sciences, National Institutes of Natural Sciences, Okazaki, Aichi 444-8787, Japan; Department of Pediatrics, Graduate School of Medicine, The University of Tokyo, Bunkyo, Tokyo 113-8655, Japan; Division of Neurology and Metabolism, Kasr Al Ainy School of Medicine, Cairo University Children Hospital, Cairo, Egypt; Department of Morphology and Genetics, Clinical Center of Medical Genetics Federal, University of São Paulo, São Paulo, Brazil; Department of Morphology and Genetics, Clinical Center of Medical Genetics Federal, University of São Paulo, São Paulo, Brazil; Centogene GmbH, 18055 Rostock, Germany; Department of Clinical Genetics, Leiden University Medical Center, 2300 RC Leiden, The Netherlands; Department of Medical Genetics, University of Alberta, Edmonton, AB, Canada; Division of Pediatric Neurology, University of Alberta, Edmonton, AB, Canada; Affiliated to the Ruth and Bruce Rappaport Faculty of Medicine Technion-Israel Institute of Technology, Genetics Institute, Carmel Medical Center, Haifa, Israel; Pediatric Neurology Unit, Carmel Medical Center, Haifa, Israel; Affiliated to the Ruth and Bruce Rappaport Faculty of Medicine Technion-Israel Institute of Technology, Genetics Institute, Carmel Medical Center, Haifa, Israel; Department of Biology, Technion-Israel Institute of Technology, Haifa 3200003, Israel; Department of Neurology, Reinier de Graaf Hospital, 2625 AD Delft, The Netherlands; Department of Neurology, Leiden University Medical Center, 2300 RA Leiden, The Netherlands; Izmir Biomedicine and Genome Center, Dokuz Eylul University Health Campus, Izmir, Turkey; Izmir International Biomedicine and Genome Institute, Dokuz Eylul University, Izmir, Turkey; Department of Paediatric Neurology, School of Medicine, Dokuz Eylul University, Izmir, Turkey; Department of Clinical Neurosciences, School of Clinical Medicine, University of Cambridge, Cambridge Biomedical Campus, Cambridge, UK; Department of Clinical Neurosciences, John Van Geest Centre for Brain Repair, School of Clinical Medicine, University of Cambridge, Cambridge, UK; CNAG-CRG, Centre for Genomic Regulation, Barcelona Institute of Science and Technology, Barcelona, Spain; Children’s Hospital of Eastern Ontario Research Institute, University of Ottawa, Ottawa, Canada; Department of Neuropediatrics and Muscle Disorders, Medical Center–University of Freiburg, Faculty of Medicine, Freiburg, Germany; Division of Neurology, Department of Medicine, The Ottawa Hospital; and Brain and Mind Research Institute, University of Ottawa, Ottawa, Canada; Department of Clinical and Movement Neurosciences, UCL Queen Square Institute of Neurology, University College London, London WC1N 3BG, UK; Institute of Medical Genetics and Applied Genomics, University of Tübingen, Tübingen 72076, Germany; Praxis für Humangenetik, Klinikum Bremen-Mitte, Bremen 28209, Germany; Department of Pediatrics, Klinikum Bremen-Mitte, Bremen 28205, Germany; Division of Genetic Medicine, Department of Pediatrics, University of Washington and Seattle Children’s Hospital, Seattle, WA 98195, USA; Department of Otolaryngology - Head and Neck Surgery, Tübingen Hearing Research Centre, Eberhard Karls University Tübingen, Tübingen 72076, Germany; Department of Otolaryngology - Head and Neck Surgery, Tübingen Hearing Research Centre, Eberhard Karls University Tübingen, Tübingen 72076, Germany; Department of Translational Genomics, King Faisal Specialist Hospital and Research Center, Riyadh 11564, Saudi Arabia; Department of Translational Genomics, King Faisal Specialist Hospital and Research Center, Riyadh 11564, Saudi Arabia; Veltischev Research and Clinical Institute for Pediatrics of the Pirogov Russian National Research Medical University of the Russian Ministry of Health, Moscow, Russia; Veltischev Research and Clinical Institute for Pediatrics of the Pirogov Russian National Research Medical University of the Russian Ministry of Health, Moscow, Russia; Mental Health Research Center, Moscow 107076, Russia; Department of Pediatric Neurology, Indira Gandhi Institute of Child Health, Bangalore, India; Department of Pediatric Neurology, Indira Gandhi Institute of Child Health, Bangalore, India; Department of Translational Genomics, King Faisal Specialist Hospital and Research Center, Riyadh 11564, Saudi Arabia; Division of Genetic Medicine, Department of Pediatrics, University of Washington and Seattle Children’s Hospital, Seattle, WA 98195, USA; Genetics and Precision Medicine Department, King Abdullah Specialized Children's Hospital (KASCH), King Abdulaziz Medical City, Ministry of National Guard-Health Affairs (MNG-HA), Riyadh, Saudi Arabia; Medical Genomics Research Department, King Abdullah International Medical Research Center (KAIMRC), King Saud Bin Abdulaziz University for Health Sciences, King AbdulAziz Medical City, Ministry of National Guard Health Affairs, Riyadh, Saudi Arabia; Institute of Medical Genetics and Applied Genomics, University of Tübingen, Tübingen 72076, Germany; Centre for Rare Diseases, University of Tübingen, Tübingen 72076, Germany; IRCCS Istituto Giannina Gaslini, 16147 Genoa, Italy; Department of Neurosciences, Rehabilitation, Ophthalmology, Genetics, Maternal and Child Health, University of Genova, Genova, Italy; IRCCS Istituto Giannina Gaslini, 16147 Genoa, Italy; Division of Membrane Physiology, Department of Molecular and Cellular Physiology, National Institute for Physiological Sciences, National Institutes of Natural Sciences, Okazaki, Aichi 444-8787, Japan; Department of Physiological Sciences, School of Life Science, SOKENDAI (The Graduate University for Advanced Studies), Okazaki, Aichi 444-8585, Japan; Department of Clinical Genetics, Leiden University Medical Center, 2300 RC Leiden, The Netherlands; Department of Neuromuscular Disorders, UCL Queen Square Institute of Neurology, University College London, London WC1N 3BG, UK

**Keywords:** ADAM22, LGI1, refractory seizures, developmental and epileptic encephalopathy

## Abstract

Pathogenic variants in A Disintegrin And Metalloproteinase (ADAM) 22, the postsynaptic cell membrane receptor for the glycoprotein leucine-rich repeat glioma-inactivated protein 1 (LGI1), have been recently associated with recessive developmental and epileptic encephalopathy. However, so far, only two affected individuals have been described and many features of this disorder are unknown.

We refine the phenotype and report 19 additional individuals harbouring compound heterozygous or homozygous inactivating *ADAM22* variants, of whom 18 had clinical data available. Additionally, we provide follow-up data from two previously reported cases. All affected individuals exhibited infantile-onset, treatment-resistant epilepsy. Additional clinical features included moderate to profound global developmental delay/intellectual disability (20/20), hypotonia (12/20) and delayed motor development (19/20). Brain MRI findings included cerebral atrophy (13/20), supported by post-mortem histological examination in patient-derived brain tissue, cerebellar vermis atrophy (5/20), and callosal hypoplasia (4/20). Functional studies in transfected cell lines confirmed the deleteriousness of all identified variants and indicated at least three distinct pathological mechanisms: (i) defective cell membrane expression; (ii) impaired LGI1-binding; and/or (iii) impaired interaction with the postsynaptic density protein PSD-95.

We reveal novel clinical and molecular hallmarks of ADAM22 deficiency and provide knowledge that might inform clinical management and early diagnostics.

## Teaser

Van der Knoop *et al.* describe the clinical features of 21 individuals with biallelic pathogenic variants in *ADAM22* and confirm the deleteriousness of the variants with functional studies. Clinical hallmarks of this rare disorder comprise progressive encephalopathy and infantile-onset refractory epilepsy.

## Introduction

Although ion channel genes represent the gene family most frequently causally related to epilepsy,^1,2^ other genes have gradually been associated with complex developmental epilepsy conditions, revealing the pathogenic role of mutations affecting diverse molecular pathways that regulate membrane excitability, synaptic plasticity, presynaptic neurotransmitter release, postsynaptic receptors, transporters, cell metabolism and many formative steps in early brain development.^3^ One of such mechanisms comprises defects in complexes formed by the secreted neuronal glycoprotein leucine-rich repeat glioma-inactivated protein 1 (LGI1) and its post- and presynaptic receptors A Disintegrin And Metalloproteinase 22 and 23 (ADAM22 and ADAM23).^4–6^ Members of the ADAMs family of transmembrane metalloproteases are implicated in cell adhesion and proteolysis.^7^ However, ADAM22 and ADAM23 are catalytically inactive and mainly act as receptors for other (glyco-)proteins, including LGI1.^8^ The resulting LGI1-ADAM22 complex forms a 2:2 hetero-tetramer and constitutes a trans-synaptic nano-architecture to regulate synapse maturation and function, particularly in the postnatal brain.^4,9,10^ Recent studies revealed a pivotal role of LGI1-ADAM22 in AMPA-type glutamate receptor-mediated synaptic transmission and hippocampal long-term potentiation (LTP) via the postsynaptic density protein, PSD-95.^10–12^ In addition to the postsynaptic membrane, ADAM22 is also expressed in axons, where LGI1-ADAM22 participates in the expression of voltage-dependent K_v_1 channels.^13,14^ In the peripheral nervous system, ADAM22 at the juxtaparanodes regulates nerve myelination through LGI4.^15^

Defects in *LGI1*, *ADAM22* and *ADAM23* have all been genetically linked to epilepsy, indicating the physiological relevance of this complex.^16^ Knock-out mice for *Lgi1*,^5,17,18^*Adam22*^19^ and *Adam23*^20^ exhibit lethal seizures in early postnatal life. In humans, heterozygous pathogenic variants in *LGI1* have been associated with autosomal dominant lateral temporal lobe epilepsy (ADLTE; OMIM #600512).^6,21^ The deleteriousness of these LGI1 variants has been ascribed to their reduced secretion or reduced ability to interact with ADAM22.^4,5,21^ Similarly, LGI1 autoantibodies that occur in limbic encephalitis inhibit the LGI1–ADAM22 interaction.^22–24^ While mutations in *ADAM23* have not been identified in human disease, Muona *et al*.^25^ reported a female case with severe infantile-onset progressive encephalopathy and intractable seizures who harbored compound heterozygous variants in *ADAM22* that compromised its function. To further establish the clinical consequences of pathogenic variants in *ADAM22*, it is imperative to identify and phenotypically characterize additional affected individuals. However, to the best of our knowledge, only one other case has been reported as part of studying a large cohort of families with Mendelian disorders.^26^

We report 19 additional cases with potentially deleterious variants in *ADAM22* identified through next-generation sequencing, of whom 18 had clinical data available, and include additional features of two previously reported cases.^25,26^ All affected individuals presented moderate-profound global developmental delay, intellectual disability, and infantile-onset epilepsy. Complementary studies in heterologous expression models confirmed the pathogenic nature of the identified variants and revealed three distinct pathogenic mechanisms.

## Materials and methods

### Case ascertainment

All affected individuals were ascertained through patient care and had been evaluated for severe epilepsy syndromes with next-generation sequencing techniques in a clinical context. A number of individuals had been identified through the collaborative SYNaPS study which is a multi-national study to investigate rare and undiagnosed neurological disorders, and were subsequently enrolled in this study. In addition, we screened a large set of exome and genome sequencing data from different diagnostic and research genetic laboratories including Centogene, GeneDx, Baylor Genetics, Invitae, 100 000 Genome Project, Queen Square Genomics, ClinVar, Decipher, DDD study, Geno2MP and many other local databases worldwide as well as using GeneMatcher.^27^ Clinical data were retrieved from medical records retrospectively. Brain MRI studies were reviewed centrally by an experienced pediatric neuroradiologist (M.S.) and clinical data as well as EEG recordings were reviewed and evaluated by a paediatric epileptologist (P.S.). For both affected individuals who have been previously described,^25,26^ we provide exhaustive additional clinical and/or histological data.

### Ethical consideration

This study has been approved by the relevant institutional review boards. Written informed consent for DNA analysis and the use of medical data for this publication was obtained from all parents or legal representatives of these children.

### Exome sequencing and bioinformatic analysis

Genomic DNA of all affected individuals and indicated family members was extracted from peripheral white blood cells and used for whole-exome sequencing (WES) (details are provided in the Supplementary material). Sequences were aligned to the Human Reference Genome (GRCh38) and their characteristics are detailed in the Supplementary material. Candidate variants were confirmed, and their segregation (if possible) was evaluated by bi-directional Sanger sequencing. Primer pairs are available upon request. RefSeq ID NM_021723.3 was used to indicate *ADAM22* variants.

### Functional studies

Functional and structural studies have been performed using well-established protocols,^4,25^ summarized in the Supplementary material. Briefly, using transiently transfected COS7 cells, the impact of the identified variants on ADAM22 protein expression levels and subcellular localization was assessed by immunoblotting and cell-surface localization studies, respectively, and the binding capacity of the different ADAM22 variants with LGI1 and PSD-95 was evaluated by co-immunoprecipitation studies. The recently published ADAM22 crystal structure (protein databank #5Y2Z) was used as a template for structural modelling of the variants.^9^ Splice site variants were evaluated using mini-gene splicing assays as described before.^28,29^

### Data availability

Because of the rarity of the disorder, individual participant data beyond those reported in this article will not be shared, to safeguard patient privacy.

## Results

### Clinical features

The clinical features available of 20 out of 21 affected individuals identified from 16 families are described in the Supplementary material, Supplementary Fig. 1 and Supplementary Table 1 and summarized in Table 1,^25,26,30–33^ including two previously described by Muona *et al*.^25^ and Maddirevula *et al*.^26^ All affected individuals presented with infantile-onset seizures [median (range) age of first symptoms 2 (0–18) months] that were refractory to treatment with anti-seizure medications or diet. Most affected individuals had focal tonic or clonic seizures, which later progressed to multifocal seizures in three individuals (Supplementary material). All affected individuals displayed moderate to profound intellectual disability and 19/20 (95%) showed delayed motor milestone attainment and remained non-verbal. Only 7 (50%) out of 14 affected individuals aged above 1.5 years at the time of assessment were able to walk. During clinical examination, 12 (60%) out of 20 affected individuals manifested profound general hypotonia in the first years of life, and 8 (42%) out of 19 exhibited spastic features (brisk tendon reflexes and/or contractures). Autistic features were present in four (80%) out of five affected individuals aged above 10 years, and four of them also exhibited self-mutilation. No consistent extra-neurological manifestations or dysmorphic features were noted. Patients P3A and P3B, both severely affected, died at the ages of 1.3 and 0.5 years due to (cardio-)respiratory failure, respectively. Patient P10 died of pneumonia at the age of 28 years. Patient P12A died at the age of 6 years from a status epilepticus and liver failure of unknown origin. Patients P13A and P13B, also severely affected, died of aspiration pneumonia and respiratory failure at the ages of 0.8 and 0.1 years, respectively. Patient P14 died in palliative care at the age of 6 months. Patient P16 died of respiratory failure with status epilepticus at the age of 2.2 years.

**Table 1:** Clinical characteristics of 20 out of 21 affected individuals with pathogenic variants in *ADAM22* and available clinical data, including an overview from the literature

Patient	Age (y)	Gender	Ethnicity	DNA variant	Protein variant	Sequence Method	Seizure onset (m)	Type	Refractory	EEG	ID/DD	Neurology	Behavioural problems	MRI
P1	12	F	Dutch	c.1915C>A	p.(H639N)	WES (trio)^30^	7.0	F, M, TC	Yes	SBA	Severe	—	ASD, SM, AB	CA, WMA^a^
P2	7	F	Dutch	c.1915C>A/large del	p.(H639N)/−	WES (trio)^30^	2.0	F > MF	Yes	H	Severe	Hypot, Spas	No	CA, WMA^a^
P3A	1.3^b^	F	Israeli Druze	c.2077-2A>C	p.(C694LfsX7)	WES^31^	0.1	M, T	Yes	SBA	Profound	Hypot	No	N
P3B	0.5^b^	F	Israeli Druze	*c.2077-2A>C*	*p.(C694LfsX7)*	Sanger par^c^	0.1	TC	Yes	MED	Profound	Hypot	No	CA, WMA
P4	7	M	America-European	c.1733C>T/c.2576+1G>C	p.(T578M) /p.(E859DfsX2)	WES^31^	0.1	F > MF	Yes	n.a.	Severe	Hypot, Spas	AB	CCA, CBA^a^
P5	1.7	M	Persian	c.1312C>A	p.(P438T)	WES^31^	1.3	F, TC	Yes	MED	Severe	Hypot	No	CA, WMA, CCA^a^
P6	19	F	Brazilian	c.1343G>A	p.(G448D)	WES^d^	2.5	F	Yes	SBA	Moderate	Hypot, Co	ASD	TS, CD^a^
P7	3	M	Armenian/Yezidi	c.2686C>T	p.(R896*)	WES^d^	18.0	F > MF	Yes	MED, H	Mild-moderate	—	ADHD	CA, WMA^a^
P8	4	M	Egyptian	c.1733C>T	p.(T578M)	WES^31^	6.0	M, TC	Yes	H	Profound	Hypot, chorea	AB	CA
P9	0.6^b^	M	Persian	c.1733C>T	p.(T578M)	WES^32^	3.0	T	Yes	MED	Profound	Hypot	n.a.	CA, CBA, DM, WMA, CCA^a^
P10^25^	28^b^	F	Finnish	c.1202G>A /c.2396delG	p.(C401Y) / p.(S799IfsX96)	WES^25^	3.0	F	Yes	G, SBA	Profound	Hypot, Spas	No	CA, WMA
P11^26^	19	M	Arab	c.2686C>T^e^	p.(R896*)	WES^26^	5.0	F	Yes	MED, SBA	Moderate	—	ASD, SM, ADHD	CBA^a^
P12A	6^b^	M	Iranian Arab	c.2686C>T	p.(R896*)	WES (m/sib)^32^	8.0	F > TC	Yes	MED > G	Moderate	—	ASD, Other	CBA, CCA^a^
P12B	4	M	Iranian Arab	c.2686C>T	p.(R896*)	WES (m/sib)^32^	18.0	MF	No	FED	None	—	Other	CBA^a^
P13A	0.8^b^	F	Arab	c.1744A>G	p.(N582D)	WES (trio)^31^	Birth	F > M	Yes	MED	Severe	Hypot, Spas	No	CA
P13B	0.1^b^	M	Arab	c.1744A>G	p.(N582D)	WES (trio)^31^	Birth	F > M	Yes	MED	Severe	Hypot, Spas	No	CA
P14	0.5^b^	F	German	c.247-21179_390+8515del /c.1421G>T	p.(L83_K130del)/p.(C474F)	WES + Sanger par^33,d^	0.1	MF	Yes	MED/H	Profound	N.a.	n.a.	DM
P15A	16	F	Turkish	c.1312C>A	p.(P438T)	WES (par/sib)	0.8	TC	Yes	MED, SBA	Severe	Spas	ASD, SM, AB	CA
P15B	8	F	Turkish	c.1312C>A	p.(P438T)	WES (par/sib)	2.0	TC, M	Yes	H	Severe	Hypot, Spas	ASD, SM, AB	CA
P16	2.2^b^	F	Indian	c.2433G>A	p.(W811*)	WES	Birth	TC, M	Yes	MED	Profound	Spas	n.a.	CA,WMA

Detailed case descriptions are in the Supplementary material and summarized in Supplementary Tables 1 and 2. AB = aggressive behaviour; ADHD = attention deficit hyperactivity disorder; ASD = (signs of) autism spectrum disorder; CA = cerebral atrophy; CBA = cerebellar atrophy; CCA = corpus callosum atrophy; CD = cortical dysplasia; Co = coordination problems; DD = developmental delay; DM = delayed myelination; FED = focal epileptic discharges; F = focal; G = generalized epileptic activity; H = hypsarrhythmia; Hypot = hypotonia; ID = intellectual disability; m = mother; M = myoclonic; MED = multi-focal epileptic discharges; MF = multi-focal; N = normal; n.a. = not available; par = parents; SBA = slowed background activity; sib = siblings; SM = self-mutilation; Spas = spasticity; T = tonic; TC = tonic-clonic; TS = temporal sclerosis; WMA = white matter alterations.

a Brain imaging centrally reassessed.

b Age at death.

c Since no DNA was available for Patient P3B due to early death, the presence of a bi-allelic pathogenic variant in Patient P3B (italics) was inferred from the identification of pathogenic variants on one allele in both parents and based on the strong similarities in clinical phenotype compared to Patient P3A within the same family (Supplementary Fig. 1).

d See Supplementary Table 1 for details on sequencing methods.

e Maddirevula *et al*.^26^ used RefSeq ID NM_016351.4 instead of RefSeq ID NM_021723.3. Large del: del 7q21.12 (87, 576, 407–87, 737, 435).

**Figure 1:**
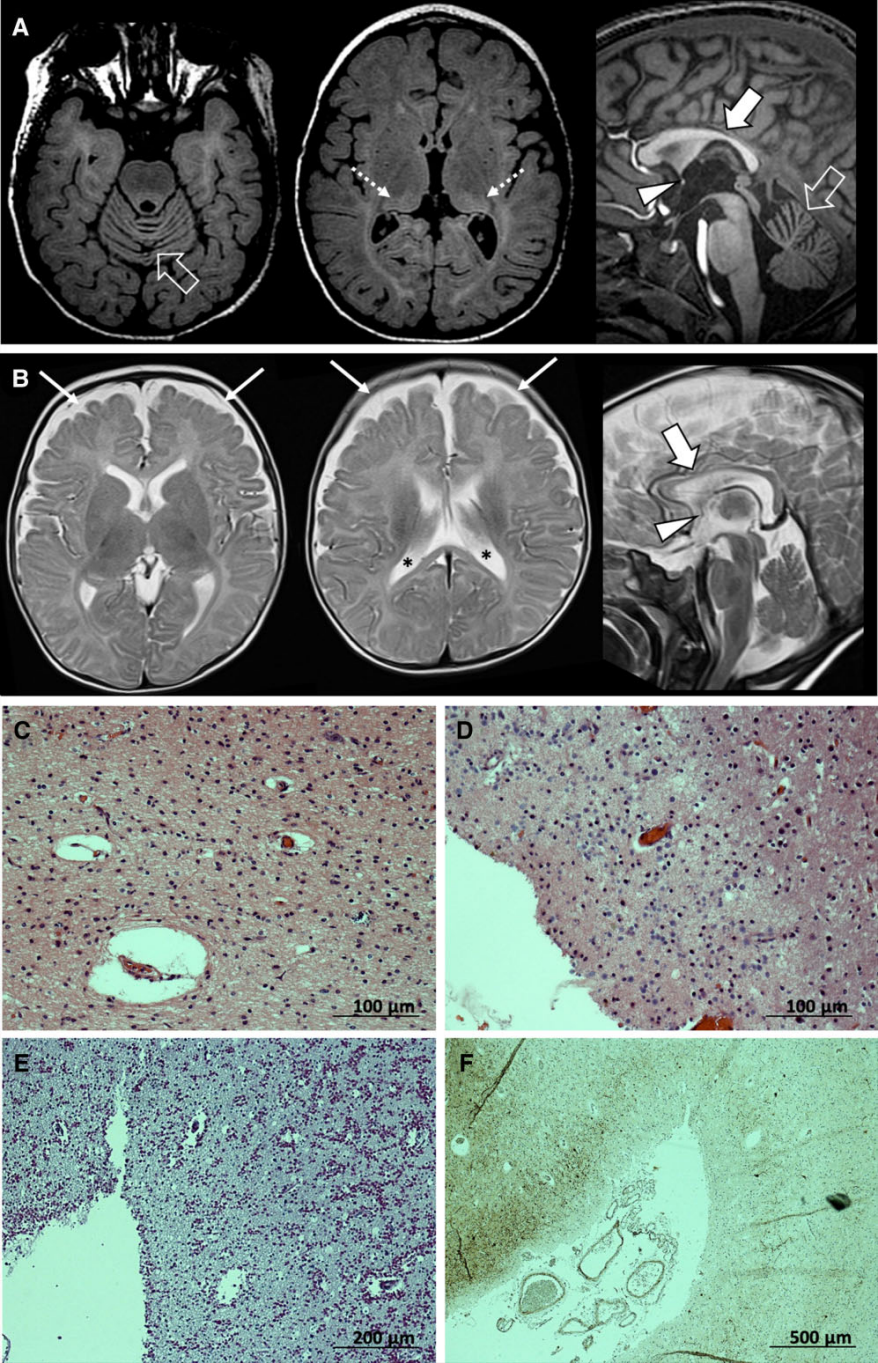
**Brain MRI and histology.** (**A** and **B**) Relevant neuroimaging features associated with *ADAM22* variants, including cerebral atrophy with enlargement of the CSF spaces (thin arrows) and lateral ventricles (asterisks), cerebellar atrophy with prevalent vermian involvement (empty arrows), corpus callosum hypoplasia/thinning (thick arrows) and anterior commissure hypoplasia (arrowheads). Additional diffuse hyperintensity of the supratentorial white matter with bilateral pulvinar involvement (dotted arrows) was noted in one subject on FLAIR images (**A**) from Patient P4 and (**B**) from Patient P5. (**C**–**F**) Post-mortem examination of brain tissue obtained from Patient P10 (deceased at the age of 28 years). (**C**) Haematoxylin and eosin-staining (×200 magnification) of the visual cortex, which showed profound atrophy and neuronal depletion with only some pyramidal cells in layers V–VI. (**D**) Haematoxylin and eosin-staining (×200 magnification) of the medial thalamus which was extremely atrophic and gliotic. (**E**) PAS staining (×100 magnification) of the frontal cortex which was very atrophic with a vast number of corpora amylacea. (**F**) Neurofilament SMI32 staining by immunohistochemistry (×40 magnification), showing the pronounced loss of neurons at the sulcal region.

Brain MRI revealed mild to moderate cerebral atrophy with reduced white matter volume in 13/20 (65%) subjects (Patients P1, P2, P3B, P5, P7-P10, P13A, P13B, P15A, P15B and P16; Fig. 1A and Supplementary Fig. 2). Cerebellar atrophy with prevalent superior vermis involvement was noted in 5/20 (25%) individuals (Patients P4, P9, P11, P12A and P12B; Fig. 1B). Corpus callosum hypoplasia/thinning was noted in 4/20 (20%) subjects (Patients P4, P5, P9 and P12A), while hypoplasia of the anterior commissure was noted in 11/20 (55%) cases (Fig. 1A and B). Two subjects (Patients P2 and P12A) presented enlarged perivascular spaces in the corpus callosum. In addition, Patient P4 presented diffuse white matter signal changes associated with bilateral pulvinar T_2_ hyperintensity (Fig. 1A), and Patient P6 had left hippocampal sclerosis with T_2_ hyperintensity of the ipsilateral anterior temporal lobe. Imaging findings were normal in 3/12 (25%) subjects scanned at ≤3 months of age (Patients P2, P3A and P14), although at least one of them showed abnormalities later in life (Patient P2).

Post-mortem brain examination of Patient P10 revealed pronounced atrophy of cortical and subcortical regions (Fig. 1C–F and Supplementary material). Extreme cortical atrophy was observed, increasing rostro-caudally in the neocortex. The hippocampi were very small, but neuronal cells were better preserved than in the cortical regions. Atrophy of the white matter and brain stem was interpreted as secondary to the cortical degeneration. The cerebellum showed less pronounced changes. The deep grey matter was quite preserved in striatum, but the thalami were very atrophic and gliotic. The neocortical atrophy, especially frontally, was quite total at the sulci, with some neurons preserved at the gyral regions.

### Genetic analyses

**Figure 2:**
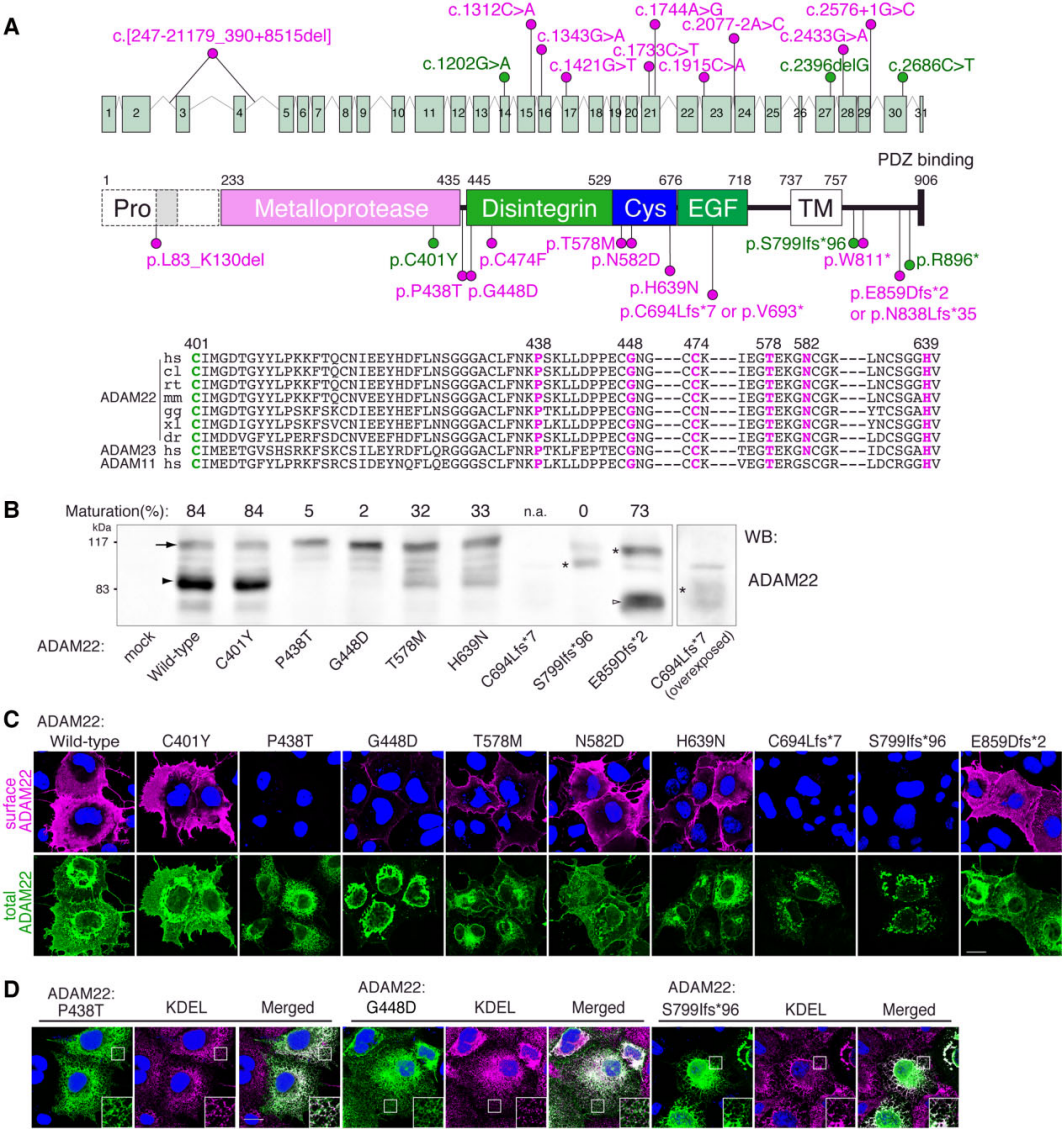
**Structural mapping and cell-surface expression of ADAM22 variants.** (**A**) ADAM22 gene structure and protein domain overview. The immature form of ADAM22 contains the N-terminal prosequence (Pro). The mature ADAM22 consists of the metalloprotease-like, disintegrin, cysteine-rich, EGF-like, transmembrane (TM) and cytoplasmic domains. The major ADAM22 isoform has a PDZ-binding motif at its C-terminus. The positions of ADAM22 variants are indicated. Missense variants are all conserved across various species and in ADAM22 family proteins (ADAM11 and ADAM23). The RefSeq ID NM_021723.3 (a long spliced form of ADAM22) is used to indicate all variants. p.C401Y, p.S799IfsTer96 and p.R896Ter are reported variants. (**B**) Maturation and expression levels of ADAM22 variants. COS7 cells were transfected with the indicated ADAM22 variants. Cell lysates were subjected to western blotting (WB) with anti-ADAM22 antibody. An arrow and an arrowhead indicate the positions of immature and mature forms of full-length ADAM22. Asterisks indicate the immature form of frame-shifted ADAM22. An open arrowhead indicates the mature form of E859DfsTer2 (indicated as E859Dfs*2). Maturation (%) was calculated by the ratio of the band intensity of the mature form to the total band intensity (mature plus immature forms). The data shown are representative of two independent experiments. (**C**) Indicated cDNAs of ADAM22 variants were transfected into COS7 cells. Cell-surface expressed ADAM22 was live-labelled by an antibody against the extracellular domain of ADAM22. To see the intracellular pool of ADAM22 expressed (total), ADAM22 was labelled with different fluorescence after the fixation and permeabilization of cells. Nuclear DNA was stained by Hoechst 33342 to distinguish transfected from untransfected cells. (**D**) P438T, G448D and S799IfsTer96 variants were predominantly localized in the endoplasmic reticulum labelled by the anti-KDEL antibody. Regions outlined with squares are magnified (large *insets*). Scale bars = 20 μm (**C** and **D**). Please note that the provided immunoblots have been cropped; full images are provided in Supplementary Fig. 6.

In the absence of explanatory genetic causes being identified with karyotyping, SNP arrays or targeted sequencing of epileptogenic candidate genes, WES was performed in all families. The results are summarized in Fig. 2A, Table 1 and Supplementary Tables 1 and 2, and described in detail in the Supplementary material. Briefly, ultra-rare homozygous or compound heterozygous variants in *ADAM22* were identified in all affected individuals, which segregated within the families (Fig. 2A, Supplementary Table 2 and Supplementary Fig. 1). Parents of affected individuals were consanguineous in 11/16 (69%) families. Of the seven different missense variants identified, c.1733C>T; p.(Thr578Met) (Patients P4, P8 and P9), c.1915C>A; p.(His639Asn) (Patients P1 and P2) and c.1312C>A; p.(Pro438Thr) (Patients P5 and P15A-B) recurred in multiple, unrelated families. Other missense variants, c.1343G>A; p.(Gly448Asp) (P6), c.1202G>A; p.(Cys401Tyr) (Patient P10),^25^ c.1744A>G; p.(Asn582Asp) (Patients P13A and P13B) and c.1421G>T; p.(Cys474Phe) (Patient P14), occurred only within one family. All identified (missense) variants affecting highly conserved residues are absent or extremely rare in heterozygous state across multiple large human variant databases (over half a million individuals) and were predicted to be deleterious by most *in silico* prediction tools (Supplementary Table 2).

The c.2686C>T; p.(Arg896*) variant, recently reported in Patient P11,^26^ was also identified in two unrelated families (Patients P7 and P12A-B). Haplotype analysis using WES data revealed a possible founder effect of the c.2686C>T; p.(Arg896*) variant in Middle-Eastern families. Another truncating variant, c.2433G>A; (p.Trp811*), was identified in Patient P16.

Patient P3A was homozygous for a c.2077-2A>C variant, abolishing the splice acceptor site of exon 24 with a predicted outcome involving exon 24 skipping [p.(Cys694LeufsTer7)]. However, *in vitro* RNA splicing studies indicated the activation of a cryptic splice acceptor site eight nucleotides downstream from the native splice site that immediately introduced a premature stop codon [r.2077_2084del, p.(Val693*)] (Supplementary Fig. 3A–C). In both cases, c.2077-2A>C variant transcripts containing premature stop codons are likely to undergo nonsense-mediated decay (NMD). Although DNA was not available, her younger sister, Patient P3B, was likely homozygous for the same variant (Supplementary material). Patient P4 was compound heterozygous for a c.1733C>T; p.(Thr578Met) variant and a c.2576+1G>C variant, affecting the splice donor site of exon 29. *In vitro* RNA splicing studies identified two abnormally spliced amplicons, with the majority of amplicons (43/44 clones, 98%) skipping of exon 29 [r.2510_2576del, p.(Asn838LeufsTer35)], and a minority (1/44 clones, 2%) activating a cryptic splice donor site in intron 29 [r.2576_2576+1ins37, p.(Glu859AspfsTer2)] (Supplementary Fig. 3D–F).

None of the affected individuals had other pathogenic/likely pathogenic variants identified in other relevant disease-associated genes.

### Functional characterization of variants identified in ADAM22

We performed functional studies in COS7 cells transfected with wild-type or mutant ADAM22 expression constructs and assessed (i) protein maturation; (ii) total protein expression; (iii) cell-surface expression; (iv) LGI1-binding; and (v) PSD-95-binding (summarized in Supplementary Table 3).

Upon overexpression in COS7 cells, wild-type ADAM22 protein was effectively processed from its pro-form (<20%, arrow) to its mature form (>80%, arrowhead; Fig. 2B). The maturation levels of Cys401Tyr and Glu859AspfsTer2 were similar to that of wild-type, whereas those of Thr578Met, Asn582Asp and His639Asn were reduced (Fig. 2B and Supplementary Fig. 4C and D). As previously described,^25^ the Ser799IlefsTer96 variant remained immature and its expression level was lower than that of wild-type, as was the case for Pro438Thr, Gly448Asp, Cys474Phe, and Leu83_Lys130del. The expression level of truncated Cys694LeufsTer7 was greatly diminished. Live-labelling of cell-surface-expressed ADAM22 showed the efficient cell-surface expression of wild-type, Cys401Tyr, Asn582Asp and Glu859AspfsTer2 ADAM22 (Fig. 2C). Compared to the wild-type, the cell surface expression of the Cys474Phe, Thr578Met and His639Asn variants were reduced. The Leu83_Lys130del, Pro438Thr, Gly448Asp, Cys694LeufsTer7 and Ser799IlefsTer96 variants were hardly expressed on the cell surface (Fig. 2C and Supplementary Fig. 4A) and were retained in the endoplasmic reticulum (Fig. 2D).

**Figure 3:**
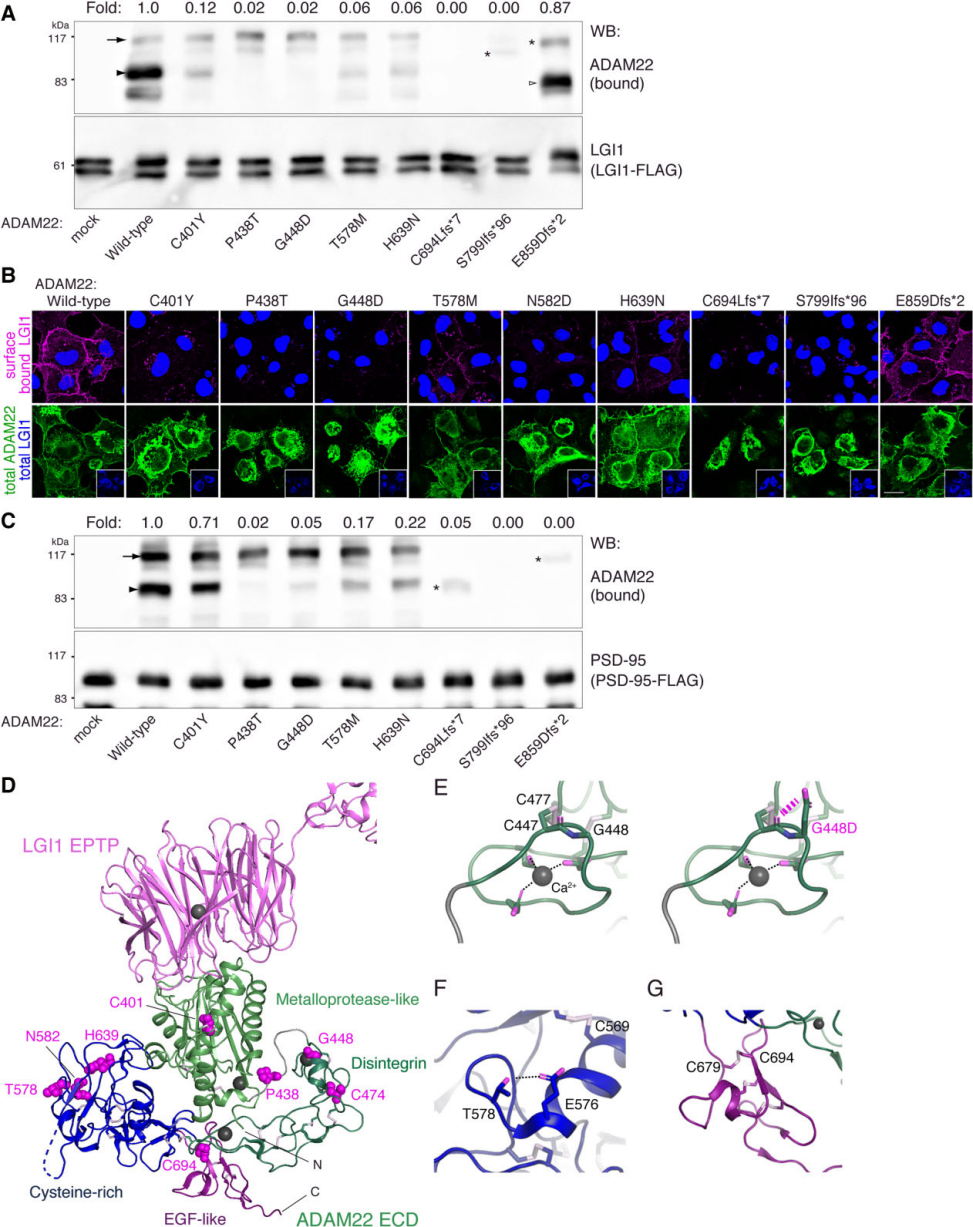
**LGI1- and PSD-95-binding activities of ADAM22 variants.** (**A**) The interaction of ADAM22 variants with LGI1-FLAG was examined by immunoprecipitation with FLAG antibody in lysates derived from COS7 cells transiently co-transfected with wild-type or indicated variant ADAM22 and LGI1-FLAG. ADAM22 variants besides E859Dfs*2 showed reduced or no binding to LGI1. Immature ADAM22 (arrow and asterisks) was often observed when overexpressed in cells and seemed to be non-specifically precipitated under the conditions. In the rodent brain lysate, immature forms are hardly detected.^4^ (**B**) LGI1-FLAG and ADAM22 variants were co-expressed and cell-surface bound LGI1 through ADAM22 was live-labelled by anti-FLAG antibody. After fixation and permeabilization of cells, protein expression of ADAM22 (total) and LGI1 (in *insets*; total) was validated. (**C**) The interaction of ADAM22 variants with PSD-95 was investigated as in **A**. E859DfsTer2 selectively lost the binding to PSD-95. Extracellular missense variants showed various levels of PSD-95 binding, according to their expression levels. Fold changes in LGI1 (**A**) or PSD-95 (**C**) binding of variants relative to the wild-type are shown. The data shown are representative of two independent experiments. (**D**) Mapping of eight ADAM22 variants on the LGI1 EPTP-ADAM22 structure. The corresponding amino-acid residues are shown. (**E**–**G**) Close-up views of G448 (**E**), T578 (**F**) and C694 (**G**). The G448D mutation causes a steric hindrance to C447 (**E**, *right*) and impairs the disulphide bond formation between C447 and C477, which supports the Ca^2+^ coordination (**E**, *left*). The T578M mutation impairs the hydrogen bond formation between T578 and E576 (**F**). The C694L mutation disrupts the disulphide bond formation between C679 and C694 (**G**). Note that provided immunoblots have been cropped; full images are provided in Supplementary Fig. 6. ECD = extracellular domain of ADAM22.

Next, we studied to what extent the identified variants affect the binding of ADAM22 to its ligand LGI1. Co-immunoprecipitation and cell-based binding studies demonstrated efficient binding of the wild-type and Glu859AspfsTer2 ADAM22 to LGI1 (Fig. 3A and B). The LGI1 binding capacity of Cys401Tyr, The578Met, Asn582Asp and His639Asn was reduced compared to the wild-type, whereas LGI1 binding was completely abrogated by the Leu83 Lys130del, Pro438Thr, Gly448Asp, Cys694LeufsTer7, Cys474Phe and Ser799IlefsTer96 variants (Fig. 3A and B and Supplementary Fig. 4B and C).

Since the Glu859AspfsTer2 variant was effectively expressed at the cell surface and did not affect LGI1 binding, we explored putative alternative pathogenic mechanisms for this variant. The Glu859AspfsTer2 variant lacks 47 cytoplasmic amino acids, including the C-terminal PDZ-binding motif (-ETSI) implicated in the binding of ADAM22 to the PDZ domain-containing protein PSD-95.^4,11^ Therefore, we examined if Glu859AspfsTer2 affects the interaction of ADAM22 and PSD-95. As reported for Ser799IlefsTer96^25^ (Fig. 3C), the Glu859AspfsTer2 variant indeed abolished the interaction with PSD-95 (Fig. 3C). The Arg896* variant, which was previously reported (Patient P11)^26^ and also identified in this study (Patients P7 and P12A-B), lacks the C-terminal 11 amino acids. Consistently, the variant was recently reported to bind to LGI1 but not to PSD-95.^10^

In addition to the ADAM22 variants identified in individuals with developmental and epileptic encephalopathy (DEE), we selected three homozygous missense ADAM22 variants (c.163C>T; p.(Leu55Phe), c.694C>T; p.(Arg232Cys), and c.2680G>A; p.(Val894Met) from the gnomAD database which contains sequencing data from presumably healthy individuals. In line with the absence of obvious clinical features, all three variants showed normal cell-surface expression, LGI1-binding, and PSD-95-binding (Supplementary Table 3 and Supplementary Fig. 5), despite being predicted as probably or possibly damaging by *in silico* prediction tools (Supplementary Table 2). Therefore, our functional assays are effective to distinguish between (likely) pathogenic and (likely) benign variants. All full western blots are presented in Supplementary Fig. 6.

Deleterious effects of some ADAM22 variants could be explained based on the protein structure of the LGI1-ADAM22 complex (protein databank #5Y2Z; Fig. 3D).^9^ The Cys401Tyr variant disrupts the disulphide bond between C394 and C401 in maintaining the LGI1-binding loop of ADAM22.^9^ In contrast, the other variants had a pronounced effect on protein expression and maturation, suggesting their defects in protein folding and stability. Indeed, the acquisition of a negatively charged Asp in the Gly448Asp variant may cause steric hindrance to Cys447 (Fig. 3E, right) and impairs the disulphide bond formation between Cys447 and Cys477 (Fig. 3E, left). The Thr578Met substitution disrupts the hydrogen bond formation between Thr578 and Glu576, and may indirectly impair the disulphide bond formation between Cys569 and Cys635 (Fig. 3F). The Cys694Leu (or Val693*) variant disrupts the disulphide bond formation between Cys679 and Cys694, destabilizing the EGF-like domain of ADAM22 (Fig. 3G). The Cys474Phe variant impairs the disulphide bond formation between Cys458 and Cys474, which supports the Ca^2+^ coordination, and potentially destabilizes the disintegrin domain of ADAM22 (Supplementary Fig. 4E).

## Discussion

We report 21 individuals with an autosomal recessive DEE characterized by moderate-profound intellectual disability, developmental delay and refractory seizures, in whom compound heterozygous and homozygous genetic variants in *ADAM22* were identified. In-depth phenotyping in 20 affected individuals allowed assessment of the core features of this ultra-rare disorder and the availability of brain tissue from a deceased patient provided a unique opportunity to describe the pathological consequences of defective ADAM22. Complementary functional studies in transfected mammalian cells confirmed that all identified variants were deleterious and interfered with normal ADAM22 function through distinct pathogenic mechanisms. Together, our findings may advance understanding of the pathogenic consequences of inactivating variants in *ADAM22* and shed light on the role of ADAM22 in human brain physiology.

Defects in the LGI1-ADAM22 complex constitute a novel epileptogenic mechanism independent of well-established channelopathies. Although numerous individuals with defective LGI1 have been reported to date, only two individuals with defective ADAM22 had been described so far.^25,26^ This study substantially extends the existing literature by reporting on 19 novel cases with compound heterozygous or homozygous pathogenic variants in *ADAM22*. All affected individuals manifested infantile-onset seizures that often progressed from focal symptoms to multifocal seizures and were refractory to different combinations of medications and dietary treatments. Moderate-profound intellectual disability and delay in (gross) motor development were common features and most affected individuals had hypotonia in early life. These characteristics are in line with those observed in a previously reported individual.^26^

On imaging, rapidly progressive cerebral atrophy has been described in two individuals with defective ADAM22.^25,26^ In the present study, brain imaging ranged from normal, especially in the early phases of the disease, to mildly-to-moderately abnormal with cerebral atrophy and reduced white matter volume observed in more than half of the subjects. These radiological findings were supported by post-mortem examination of patient-derived brain tissue, showing pronounced cortical and subcortical atrophy with loss of neuronal cells. Brain atrophy in LGI1-ADAM22 complex deficiency may result from different mechanisms, including impaired functional maturation of postnatal synapses and seizure-related brain damage, as frequently described in other early-onset epileptic encephalopathies.^11,34^ This study widens the imaging spectrum associated with ADAM22 variants, including cerebellar atrophy with prevalent vermian involvement, corpus callosum and/or anterior commissure hypoplasia and cerebral white matter signal changes. These findings suggest that LGI1-ADAM22 complex might have additional roles in brain development.

Naturally occurring variants in *ADAM22* are rare in the healthy population, with only two exceeding an allele frequency of 0.05. In contrast to a selection of such variants, complementary functional studies confirmed the deleteriousness of all variants identified within our cohort. These studies suggested that mutations in ADAM22 classify at least three distinct pathogenic mechanisms (summarized in Supplementary Table 3). First, defective maturation and reduced cell-surface expression of ADAM22 may reduce LGI1 binding, as was observed for the Pro438Thr, Gly448Asp, Ser799IlefsTer96, and Cys694LeufsTer7 variants. Second, the Cys401Tyr variant showed a selective defect in LGI1 binding, which aligns with recent structural analyses.^9^ Finally, the Glu859AspfsTer2 retained normal LGI1 binding and surface expression but selectively lost the ability to interact with PSD-95 as was recently reported for the Arg896* variant.^10^ Given the phenotypic features of the affected individuals with Glu859AspfsTer2 or Arg896* were similar, although less severe, to those observed in individuals with fully inactivating ADAM22 variants, these observations may indicate that the establishment of this interaction is indispensable for normal ADAM22 function. Consistently, a recent study showed that loss of the ADAM22 PDZ ligand (i.e. C-terminal five amino acids) causes lethal epilepsy around 2–8 months of age in mice and that the LGI1–ADAM22–PSD-95 interaction is a key player in the trans-synaptic nanoarchitecture for precise synaptic transmission.^10^ Pathogenic genetic variants in *DLG4*, encoding PSD-95, have been recently identified in subjects with intellectual disability,^35,36^ highlighting the importance of PSD-95 in human brain development. Notably, the Cys401Tyr, Thr578Met and His639Asn variants retained some residual LGI1- and PSD-95-binding, which may correspond to the somewhat less severe phenotype observed in individuals harbouring these variants compared with individuals homozygous for fully inactivating variants [i.e. p.(Cys694LeufsTer7)], who died in early infancy. This corresponds to the early lethality observed in *Adam22* knock-out mice.^19^ Although not functionally evaluated, the Trp811* variant was predicted to undergo nonsense mediated decay and abrogate C-terminal PSD-95 binding, together resulting in severe loss of ADAM22 function. This may explain the severe and lethal phenotype in P16. Together, these findings suggest the presence of a genotype-phenotype correlation. It should be noted that all functional studies were performed in transfected mammalian cells, and hence pathogenic mechanisms may well differ from the *in vivo* situation. Therefore, one cannot exclude the possibility that the observed effects in functional studies might be epi-phenomena of a unifying effect present in neurons or neuronal aggregates, not apparent in COS7 experiments. Nevertheless, our set of functional studies reliably distinguished pathogenic from benign variants, and, for at least some variants, outperformed publicly available *in silico* tools that predict pathogenicity, while also adding information on potential distinct pathogenic mechanisms.

Some affected individuals achieved partial seizure control on topiramate and benzodiazepine treatment. Since many commercially available anti-epileptic drugs, dietary approaches, such as the ketogenic diet, and combinations thereof have been tried and proven to be ineffective in achieving full seizure control in (most) individuals with ADAM22 insufficiency, it appears that there are no currently approved treatment options available that would specifically be effective in this disorder. Yet, the defective intracellular trafficking of ADAM22 variants (e.g. Pro438Thr and Gly448Asp) is similar to that of ΔPhe508-CFTR in cystic fibrosis and LGI1 mutants in ADLTE.^21,37^ Chemical correctors including chemical chaperones and proteostasis regulators effectively correct defective CFTR and LGI1 folding and increase their cell-surface expression and secretion, respectively.^21,38,39^ Therefore, it might be possible that some ADAM22 mutant proteins can be chemically corrected. In addition, gene therapy approaches may offer a therapeutic solution, providing target cells within the brain can be properly, and timely, targeted.

One may wonder why ADAM22-related encephalopathy shows much severer clinical patterns than LGI1-related ADLTE^6^ and autoimmune-mediated LGI1 encephalitis.^40,41^ All individuals with ADAM22-related encephalopathy have biallelic *ADAM22* variants and their parents with the monoallelic variant do not show any symptoms. In contrast, monoallelic variants in *LGI1* cause therapy-responsive, mild epilepsy, but individuals with biallelic *LGI1* variants have not been reported, probably due to their lethality. The different gene-dosage sensitivities between *ADAM22* and *LGI1* might be explained by possible different degrees of compensational or redundant expressions of their family proteins, LGI2, 3, 4 and ADAM23, 11.^5,42,43^ It suggests that the clinical severity depends on the amount of residual LGI1-ADAM22 protein complex. In the case of LGI1 antibody encephalitis, the clinical symptoms depend on how much and where in the brain LGI1 autoantibodies are present to reduce the LGI1-ADAM22 protein complex, and therefore removal of LGI1 autoantibodies by immunotherapy could be mostly effective.^40,41^ Further studies are required to understand the mechanism for different clinical patterns of the three diseases.

Our study had limitations inherent to its retrospective design. Generally, such studies are prone to the collection of incomplete datasets, possibly resulting in selection bias. Indeed, most parameters have not been obtained in all individuals, resulting in missing data. Moreover, we noticed that several features were not consistently present in all individuals, including abnormalities on brain MRI. This might be explained by differences in age at assessment in residual ADAM22 function and in other genetic factors that may modulate brain development and function.

This study further supports that inactivating variants in *ADAM22* cause human disease and give rise to severe developmental delay and infantile-onset epilepsy. Our detailed phenotypic characterization and molecular studies substantiate knowledge on this ultra-rare DEE. Identification and phenotyping of additional individuals with ADAM22 deficiency and longer follow-up will help to further delineate this disorder and optimize its clinical management.

## Supplementary Material

awac116_Supplementary_Data

## Abbreviations

ADAM: A Disintegrin and Metalloproteinase
DEE: developmental and epileptic encephalopathy
PSD: postsynaptic density
WES: whole-exome sequencing
