# Supplementary material for: Biallelic ADAM22 pathogenic variants cause progressive encephalopathy and infantile-onset refractory epilepsy
Source: Brain. 2022 Apr 4;145(7):2301–12. doi: 10.1093/brain/awac116 (PMC9337806; doi:10.1093/brain/awac116)

Supplementary Material

1. Supplementary Materials and Methods
2. Supplementary Results
3. Supplementary Tables
4. Supplementary Figures

Supplementary Materials and Methods

Exome sequencing and variant calling

Genomic DNA of the affected individuals and indicated family members was extracted from peripheral white blood cells and used for whole exome sequencing (WES) according to previously described methods (see **Table 1** for detailed references**).** Sequences were aligned to the Human Reference Genome (GRCh37) and were further processed as described before. In principle, WES was performed on the patient-parent trio, unless otherwise indicated. The RefSeq ID NM_021723.3 (a long spliced form of *ADAM22*) was used to indicate all identified variants.

Functional studies

In vitro splice assay

RNA studies of the c.2077-2A>C and c.2576+1G>C splice variants were performed as described before with minor adaptations (1). Testing of the c.2077-2A>C variant ensued by amplifying a 1,664 bp genomic region from control and patient DNA samples that included exon 24 (114 bp), as well as 550 bp and 1,000 bp from flanking 5´ and 3´ intronic sequence, respectively. Amplification ensued with primers containing a *XhoI* and *BamHI* restriction site (forward primer: 5'-aattctcgagTTCCCCTGACCCTGACATAG-3' and reverse primer: 5'-attggatccCTTCAAGACCTAGCATGTCTGC-3'), respectively. Amplification of the c.2576+1G>C variant ensued by amplifying a 1,967 bp genomic region from the patient and control DNA samples that included sequence from intron 27 (651 bp), exon 28 (101 bp), intron 28 (352 bp), exon 29 (67 bp), and intron 29 (796 bp). Primers containing *XhoI* and *BamHI* restriction sites were used (forward primer: 5'-aattctcgagAATAGCAGGTGGGCTCAATG-3'

and reverse primer: 5'-attggatccGGCAAGTTCAGTCCAAAAGG-3'), respectively. After PCR amplification and clean-up, restriction enzyme digestion of the PCR fragment and pSPL3 exon trapping vector was performed prior to ligation between exon A and exon B of the linearized pSPL3-vector. The vector was transformed into DH5α competent cells (NEB 5-alpha, New England Biolabs) that were plated and incubated overnight. The wild-type and mutant-containing vector sequences were Sanger sequence confirmed.

Vectors each containing the homozygous mutant and wild-type sequence at the c.2077-2 and c.2576+1 positions were transfected into HEK293T cells (ATCC) at a density of $2x10^5$ cells per mL. 1 µg of the respective pSPL3 vector was transiently transfected using 3 µl of FuGENE 6 Transfection Reagent (Promega). An empty vector and transfection negative reaction were included as controls. The transfected cells were harvested 24 hours after transfection. Total RNA was prepared using miRNAeasy Mini Kit (Qiagen). Approximately 1 µg of RNA was reverse transcribed using a High Capacity RNA-to-cDNA Kit (Applied Biosystems) following the manufacturer's protocols. The cDNA was PCR amplified using vector-specific SD6 forward (5'-TCTGAGTCACCTGGACAACC-3') and SA2 reverse (5'-ATCTCAGTGGTATTTGTGAGC-3') primers. The amplified fragments were visualized on a 2% agarose gel and Sanger sequenced. cDNA amplicons from the patient c.2576+1G>C variant were cloned and sequence-confirmed following standard protocols for the TA cloning Dual Promoter with pCRII Kit (Invitrogen).

Plasmid Construction

The generation of the pCAGGS: human LGI1-FLAG, human ADAM22 (wild-type), ADAM22 C401Y (c.1202G>A), and S799IfsTer96 (c.2396delG) expression constructs has been described previously (2). ADAM22 L83_K130 del (c.247-21179_390+8515del, deletion of exon 3 and 4), P438T (c.1312C>A), G448D (c.1343G>A), C474F (c.1421G>T), T578M

(c.1733C>T), N582D (c.1744A>G), H639N (c.1915C>A), C694LfsTer7 (c.2077-2A>C, skipping of exon 24), E859DfsTer2 (c.2576+1G>C), L55F (c.163C>T), R232C (c.694C>T), and V894M (c.2680G>A) were generated by the standard PCR method using primers listed in **Supplementary Table 4**. All PCR products were analyzed and confirmed by DNA sequencing.

Antibodies

The antibodies used in this study included: a guinea pig polyclonal antibody to LGI1 (LGI1-GP-Af510, Frontier Institute), mouse monoclonal antibodies to FLAG (F3165, Sigma-Aldrich), ADAM22 (N46/30, NeuroMab), KDEL (ADI-SPA-827-D, Enzo) and PSD-95 (MA1-045, ThermoFisher Scientific). Rabbit polyclonal antibody to ADAM22 was raised against GST-mouse ADAM22 (amino acids, 444-526), corresponding to the extracellular disintegrin domain as described (3).

Cell culture and transfection

COS7 cells (ATCC, CRL-1651) were cultured in 10% fetal bovine serum (Sigma-Aldrich) supplemented Dulbecco's modified Eagle medium (DMEM). For cell-surface binding assays, cells were seeded onto poly-d-lysine 12-mm cover slips in a six-well cell culture plate (2 x 10^5 cells/well) and co-transfected with LGI1-FLAG and wild-type (WT) or indicated variant ADAM22 (1 μg plasmid each) with Lipofectamine plus (Thermo Fisher Scientific). For immunoprecipitation studies, cells were seeded onto 6-well plates without coverslips and transfected with LGI1-FLAG or PSD-95-FLAG together with ADAM22 variants.

Cell-surface binding assay

Cell-surface binding assays were performed according to well-established protocols (2). Briefly, 36 h after transfection, COS7 cells were washed with DMEM and surface-expressed ADAM22 or surface-bound LGI1 were "live-labeled" for 30 min at 37 °C with antibodies

directed against the extracellular epitope of ADAM22 and FLAG, respectively. Cells were subsequently fixed with 2% paraformaldehyde/120 mM sucrose/100 mM HEPES (pH 7.4) at room temperature for 20 min and blocked with PBS containing 10 mg/ml bovine serum albumin for 10 min on ice. The fixed cells were incubated with Cy3-conjugated secondary antibody. Then, the cells were permeabilized with 0.1% Triton X-100 for 10 min, blocked with PBS containing 10 mg/ml BSA, and stained with mouse or rabbit anti-ADAM22 antibody, followed by Alexa488-conjugated secondary antibody. In Fig. 3B, total LGI1-FLAG protein was also stained after cells were permeabilized, followed by staining with Alexa 647-conjugated secondary antibody. Then, cells were stained by with Hoechst dye (33342, Thermo Fisher Scientific). Fluorescent images were acquired by confocal microscopy (Carl Zeiss LSM5 Exciter; Carl Zeiss).

Immunoprecipitation

Immunoprecipitation studies were performed as previously described (2). Briefly, 48 h after transfection, COS7 cells were washed with PBS and subsequently lysed with buffer A [20 mM Tris-HCl (pH 8.0), 1 mM EDTA, 1.3% Triton X-100 and 50 µg/ml PMSF]. The lysates were cleared by centrifugation at 10,000 g for 5 min at 4°C. LGI1-FLAG or PSD-95-FLAG was precipitated with FLAG-M2 agarose (Sigma-Aldrich) for 1 h, washed with buffer B [20 mM Tris-HCl (pH 8.0), 1 mM EDTA, 100 mM NaCl, 1% Triton X-100 and 50 µg/ml PMSF], and eluted with buffer B containing 0.25 mg/ml FLAG peptide (2, 3). The immunoprecipitates were separated by SDS-PAGE and gels were subjected to Western blotting according to standard protocols. LGI1-FLAG, PSD-95-FLAG and ADAM22 were probed with antibodies indicated in the respective figures. Chemical luminescent signal was detected and analyzed with the FUSION Solo system (Vilber-Lourmat).

Structural modeling

The PYMOL molecular viewer (http://www.pymol.org) was used to view the structural 3D model of ADAM22 variants. Crystal structure of LGI1 EPTP-ADAM22 complex (PDB, 5Y2Z) was used as the template (4).

Supplementary Results

Clinical features

Family 1

Patient 1 (P1), currently 12 years, was born at term after an uncomplicated pregnancy and vacuum delivery at 40 weeks of gestation. Her birth weight was 3,275 grams (-0.4 SD). She is the only child of heathy non-consanguineous parents of Caucasian descent, with no familial history of encephalopathy or epilepsy. The first 2 years she suffered from gastro-esophageal reflux. At 7 months of age, she first presented with seizures, characterized by focal tonic and myoclonic attacks. The EEG showed right temporal focal epileptiform discharges and a normal background pattern. Seizures were responsive to treatment with a combination of valproic acid and levetiracetam.

By the age of 3 years, vision and hearing had been extensively evaluated which did not reveal any abnormalities. By this age, she exhibited autistic features, including stereotypic behavior, which limited her development and social interactions. At physical examination she had normal body measurements (**Supplementary Table 1**). She had medial flaring of the eyebrows, downturned corners of the mouth with a cupids bow of the upper lip. She had a slightly low hanging columella. No other abnormalities were noted.

Neuropsychological assessments at the age of 2.5 and 5.7 years revealed profound intellectual disability with stable age-equivalent scores ~9 months on the cognitive and language subscales of the Bayley Scales of Infant Development (BSID)-II. Her gross motor development was adequate but she lacked behind in her fine motoric skills. She did not develop speech but is able to make herself clear by signs and facial expression.

She manifests severe sleeping problems, in particular an impaired day-night rhythm and decreased duration of REM-sleep. Treatment with clonidine had marginal positive effects.

During last follow-up at the age of ~10 years, she was able to walk and climb. Although she was still non-verbal, she was able to address simple questions through computer-assisted and non-verbal communication. She exhibited pronounced behavioral problems, including autistic features, aggressive behavior and incidental auto-mutilation. Evaluation of cognitive and language abilities showed no further development, nor decline compared to previous assessments.

Brain MRI at the age of 8 months revealed mild cerebral atrophy with reduced white matter volume (**Fig. 1A)**. Extensive metabolic analysis showed no abnormalities. A 250K SNP-array showed a normal female profile. Targeted DNA-analysis of *MECP2*, *CDKL5*, *TCF4*, *MEF2C*, *SCN1A*, *SCN2A*, *PCHH19*, *UBE3A* and methylation of chromosome 15q11 did not reveal abnormalities. Targeted massive parallel sequencing of 126 genes involved in epilepsy only revealed a maternally inherited *NRXN1* variant. This gene is involved in a recessive form of Pitt-Hopkins syndrome. As no variants were found on the other allele, this variant is considered not to be the cause of the epilepsy in this patient.

Family 2

Patient 2 (P2), currently 7 years of age, was born to non-consanguineous parents of Caucasian descent at a gestational age of 42+1 weeks after an uncomplicated pregnancy. She is known to have a congenital Horner syndrome. Her birth weight was 3,930 grams (0.5 SD). At the age of two months she first developed gazing moments, followed by focal tonic seizures at the right side, originating from the left temporal lobe for which oxcarbazepine was started. In the first 8 months of life, seizures were frequent especially during viral infections resulting in several hospital admissions for treatment of status epilepticus. Clobazam was added to her medication. At the age of 9 months, she developed infantile seizures with hypsarrhythmia on the EEG, necessitating treatment with ACTH, vigabatrin and nitrazepam and ketogenic diet to achieve seizure control. From the age of 4 years, she became seizure-free with a treatment regime of clobazam, topiramate, valproic acid, a ketogenic diet, and midazolam (on indication). At the age of 6 years all anti-epileptic drugs and the ketogenic diet could be discontinued. Seizures re-occurred due to pain after a femur fracture. Control of seizures was only achieved after topiramate was added to the medication that had been re-installed (valproic acid, levetiracetam, clobazam, clonazepam, and midazolam perfusor).

Although development was relatively normal in the first months of life, developmental decline was noted from the age of 10 months. From the first years of life, she manifested hypotonia and exhibited a severe delay in motor development. At the age of 5.5 years, she was able to roll over, sit independently and pivot, but was unable to crawl, walk without support, or eat independently. Although she was able to produce different sounds, she was non-verbal.

She displays several dysmorphic features, including deep set eyes, deep nose bridge, frontal bossing, tapering fingers, fetal finger pads, and ptosis and miosis of the left eye (congenital Horner). She has a good general health. Body weight, height, and head circumference were within normal range at last measurement (age 7y; **Supplementary Table 1**).

Diagnostic work up included brain MRI performed at the age of 3 months which showed no abnormalities. In particular, myelination was normal, and signs of cortical dysplasia or atrophy were absent. At 2.5 years, brain MRI showed enlargement of the cerebral subarachnoid spaces, especially at the frontal level, with reduced white matter volume and T2/FLAIR signal alterations at the level of fronto-temporal white matter. The perivascular spaces at the level of the splenium of the corpus callosum were increased in size. Magnetic resonance spectroscopy (MRS) showed no increased lactate in the basal ganglia. Metabolic screening in urine, plasma and cerebral spinal fluid (CSF) did not show any abnormalities. A SNP array revealed the presence of a 161.0 kb deletion on Chr7q21 (87,576,407-87,737,435), which contains only one coding gene (*ADAM22*) and was inherited from the healthy mother. In addition, a 233.0 kb large duplication on Chr 9q34 was found, which contains only one coding gene (*CACNA1B*). In addition to the healthy father, this variant was also found once among 6533 healthy controls and is thus considered to be a benign variant.

Family 3

Patient 3A (P3A) was born at term to consanguineous parents (first cousins) of Israeli Druze descent. Prenatal examinations identified a clenched left hand at 16 weeks of gestational age, a peri-membranous ventricular septal defect, and low abdominal circumference (1st percentile) at 31 weeks of gestational age. Several days after birth she started to manifest frequent episodes of refractory seizures, which started as clusters of myoclonic jerks, and later also included tonic seizures. Seizures were frequent (several episodes per day), with clustering and several episodes of status epilepticus have been documented. The initial EEG showed no abnormalities, but a subsequent EEG examination at the age of 3 months showed some focality with right spikes and normal background activity. Despite different treatment approaches, seizures remained

refractory, and only a partial response was achieved with a combination of ketogenic diet, topiramate, phenobarbital and clonazepam.

At last evaluation at the age of 11 months old, she had profound intellectual disability and did not attain early developmental milestones. She exhibited severe hypotonia and spontaneous movements were absent. She had recurrent vomiting, prompting Nissen fundoplication and was fed by gastrostomy.

Skeletal muscle biopsy showed grossly normal morphology with small groups of atrophic (type 1) fibers, consistent with neurogenic atrophy. Brain MRI at the age of 3 months was normal. Chromosomal micro-array analysis was normal and therefore whole exome sequencing was conducted.

She died at the age of 1.3 years due to cardiorespiratory failure.

To unravel the cause of the observed clinical features, chromosomal micro-array analyses was performed, which showed no abnormalities. Therefore, exome sequencing was deployed.

In retrospect, the symptoms of P3A were very reminiscent to those of her second cousin (P3B), who died at the age of 6 months due to respiratory failure. P3B was born at term after a pregnancy complicated by polyhydramnios and intra-uterine growth restriction (birth weight 1,847 g; <P3). Prenatal examinations at 24 weeks of pregnancy had also revealed clenched hands and rocker bottom feet. Other congenital abnormalities were camptodactyly and cutaneous hemangioma over the forehead, left eyelid and nose.

Starting from day two after birth, P3B presented recurrent episodes of tonic-clonic seizures. An EEG at the age of <3 months showed paroxysmal discharges with epileptiform template (periodic lateralized epileptiform discharges PLEDs) with slow peaked waves mainly over the right hemisphere, on the background of multifocal epileptic activity. Despite multiple

medication regimes and ketogenic diet, seizures remained refractory to treatment and only partial control was achieved.

The clinical manifestations of P3B were very similar to those of P3A, including profound intellectual disability, severe delay of motor development, hypotonia and muscular atrophy. P3B did not reach early developmental milestones and was tube fed. Transthoracic ultrasonography examinations revealed diaphragmatic paralysis. Over time, she developed a hypertrophic left ventricle with mild left ventricular outlet obstruction. Liver biopsy and metabolic screening were normal. Brain MRI at the age of 4 months was reported to show cerebral atrophy with reduction of the white matter volume. MRS was normal. Original imaging data were not available for review. Although DNA of the patient was not available for whole exome or targeted sequencing, available karyotyping analysis were normal.

Family 4

Patient 4 (P4), currently seven years of age, was born to non-consanguineous parents of Caucasian descent after a pregnancy complicated by exposure to tobacco, alcohol, and drugs. From day two after birth, he presented seizures that usually initiate with clutching of the hands and bending of the arms, associated with apneic episodes, followed by jerks at one side or the other, and sometimes both. Seizure episodes are frequent (30-40 per day) and multiple periods of status epilepticus have been reported since the age of five months. Occasionally, subsequent Todd's paralysis was observed in the postictal phase. Seizures were refractory and treatment with different (combinations of) anti-epileptic drugs (currently on levetiracetam, lacosamide, clobazam, oxcarbazepine, cannabidiol (CBD) oil and vitamin supplementation (Airborne)) and vagal nerve stimulation were not effective. Modified Atkins diet further worsened seizures. After introduction of CBD oils alertness and seizure control improved with a notable reduction in status epilepticus frequency.

At last evaluation, he had severe intellectual disability and a pronounced delay in motor development. He was unable to sit unsupported and has never walked. Although he was able to communicate with sounds and gestures, he remained non-verbal. Severe generalized hypotonia, hyperreflexia, and contractures (predominantly in the lower limbs) were noted. Brain MRI at the age of 3 years showed diffuse white matter signal alterations, corpus callosum hypoplasia, bilateral posterior thalamic signal changes, and cerebellar atrophy with prevalent vermian involvement. He is otherwise in good general health with a normal body weight and height and no apparent abnormalities in peripheral organs. His biological sister had experienced self-resolving absence seizures in childhood.

In pursuit of finding a cause for the observed phenotype, whole exome sequencing was performed.

Family 5

Patient 5 (P5), currently 20 months of age, was born at term to consanguineous parents (**Supplementary Fig. 1**) of Persian descent after an uncomplicated pregnancy. He first presented seizures at day 40 after birth. At first, he had multiple focal tonic-clonic seizures (right side of the body) on a daily basis, lasting for minutes to an hour, without postictal symptoms. Later in life, seizures evolved to generalized tonic-clonic seizures. Three status epilepticus have been documented. EEG examinations showed multifocal paroxysmal activity over the central and posterior regions of both hemispheres. Seizures were difficult to control, despite the combined use of primidone, clonazepam, and acetazolamide.

At present, he manifests hypotonia, and exhibits severe intellectual disability and delay in motor development. Verbal speech is absent. Brain MRI at the age of 4 months showed mild cerebral atrophy with reduced white matter volume and thinning of the corpus callosum.

Family 6

Patient 6 (P6), currently 19 years of age, was born at term, the second child of consanguineous parents (second cousins) of Caucasian descent after an uncomplicated pregnancy. She first presented seizures by the age of 2.5 months. Until 1 year old, she was admitted to the ICU more than 8 times to manage recurrent seizures despite anti-epileptic treatment. She manifested global developmental delay, without neurological regression. At 7 years old, she was diagnosed with autism spectrum disorder. At present, under treatment with valproic acid, topiramate and clobazam, she has focal seizures (jerking facial movements that last less than 1 minute) re-occurring every 15-20 days. Her last ICU admission occurred at the age of 18 years, when she developed epileptic status during an infectious condition. Although she has been treated with different anti-epileptic drug regimes, she has never been seizure-free for more than one month.

At last evaluation (aged 19 years), she was in good general health. She has moderate-severe intellectual disability and attained several motor skills, including crawling (at 9 months) and independent walking (at 20 months). She is also able to grab objects and eat with minimal assistance. She is toilet trained but needs help for most self-care activities. Although she is able to maintain body posture, general hypotonia and impaired coordination was noted.

Diagnostic work up included metabolic screening in urine and plasma without significant abnormalities at 7, 9 and 10 years of age. Brain MRI at age of 7 and 10 years showed left hippocampal sclerosis and T2 hyperintensity of the ipsilateral anterior temporal lobe white matter, in keeping with an associated focal cortical dysplasia. During her follow-up, she was submitted to a G-banding karyotype and MS-MLPA for Angelman Syndrome with normal results. A SNP array revealed the presence of 18 regions of loss of heterozygosity. As these findings could not explain the observed phenotype, exome sequencing was performed at 16 years.

Family 7

Patient 7 (P7), currently 3 years of age, was born at a gestational age of 40 weeks after an uncomplicated pregnancy. The boy was the second child of healthy consanguineous parents (**Supplementary Fig 1**) of Yazidian descent. Family history examination revealed isolated speech delay in his older brother and developmental delay in his maternal cousin (unavailable for examination and testing).

At birth, his weight was 3,150 g and his length was 59 cm. He reached his early developmental milestones on time: developed head control at 3 months, sat unsupported at 7 months, started crawling at 7 months, walked unsupported at 12 months. At the age of 14 months, he started to use words, but did not speak phrases. At the age of 18 months, he gradually started to present frequent seizures with clustering and lost his speech and communication abilities. Despite different treatment approaches for 1.5 years, seizures remained refractory, but recently partial improvement was reached with the combination of clonazepam and topiramate.

At last evaluation at the age of 3 years old, the boy was overweight and had increased subcutaneous fat, but a normal height (98 cm, +0.7 SD) and head circumference (49.5 cm, -0.1 SD). He displayed few dysmorphic traits: large auricles and deeply set eyes. He had moderate to severe intellectual disability with absence of expressive speech, but he was able to understand simple instructions. He experienced behavioral problems, including severe hyperactivity and impulsiveness. Motor development was not impaired as he walked independently and could run.

On EEG the main rhythm with a frequency of 5-7 Hz and an amplitude of up to 50-100 µWb was fragmented, disorganized by groups of theta waves with frequency of 4-5 Hz. There was epileptiform activity in the left hemisphere.

Brain MRI performed at the age of 2 years showed mild cerebral atrophy involving the frontal regions with minimal reduction of the white matter volume.

Family 8

Patient 8 (P8), currently 4 years of age, was born after 36 weeks of pregnancy to consanguineous parents (**Supplementary Fig. 1**) of North-African descent after an uncomplicated pregnancy. At 6 months, he first presented tonic-clonic seizures, which were refractory to treatment. EEG examinations showed hypsarrhythmia.

At present, he manifests hypotonia and choreic movements, and exhibits severe intellectual disability and delay in motor development. He is still unable to sit or walk unsupported and verbal speech is absent. Brain MRI at the age of 1 year showed mild cerebral atrophy. The diagnostic work up included basic laboratory investigations, amino acid and acylcarnitine profile, urine organic acid profile as well as very long chain fatty acids, which were all unremarkable. We also measured guanidinoacetic acid and creatine/creatinine ratio, the results of which were compatible with (X-linked) creatine deficiency. To identify the disease-causing gene, exome sequencing was performed. Yet, no pathogenic variants were detected in the coding regions or exon-intron splice junctions of the *SLC6A8*, *GAMT*, or *GATM* genes.

Family 9

Patient 9 (P9), currently 6 months of age, was born to consanguineous parents (**Supplementary Fig. 1**) of Arabic descent after an uncomplicated pregnancy. At the age of 3 months, he first presented tonic seizures, which were refractory to treatment. EEG examinations showed multifocal sharp waves over the central regions within a diffusely disorganized background activity.

At present, he manifests hypotonia, and exhibits severe intellectual disability and delay in motor development. Brain MRI performed at 3 months of age showed delayed myelination with normal brain volume. Follow-up brain MRI performed at the age of 4 months revealed rapid progression of cerebral and cerebellar atrophy with reduced white matter volume and thinning of the corpus callosum (**Fig. 1A**).

Family 10

Patient 10 (P10) has been previously described in full detail ((2) and **Table 1**). Briefly, at three months of age she first experienced seizures. The seizures started with focal symptoms and by the age of two years evolved to generalized seizures. Initial EEG showed focal spikes and decreased background activity and later showed generalized spikes initiating from the left side with abnormal background activity. Seizures were refractory and treatment with barbiturates, phenytoin, carbamazepine, valproic acid in different combinations were ineffective.

Development appeared normal up to the age of 2 months. By then, she started to manifest episodic apneas, which was followed by regression of social abilities (loss of smiling and eye contact) and global developmental delay became apparent. At 2 years, she had profound intellectual disability and physical examination revealed generalized hypotonia, brisk tendon reflexes, absence of spontaneous movement and microcephaly. Brain MRI at 11 years showed severe cerebral atrophy with reduced white matter volume. Electroretinography was normal, and ophthalmologic examination showed no abnormalities at 2 years of age, but mild atrophy of the optic nerve at 4 years and 10 months.

Neuropathy, ataxia, retinitis pigmentosa syndrome point mutation analysis and mitochondrial DNA Southern blotting gave normal results. Therefore, exome sequencing was deployed.

At the age of 28 years, she died because of pneumonia.

Family 11

Patient 11 (P11) has been previously described in full detail ((5) and **Table 1**). Briefly, P11, currently 18 years, was born at term to consanguineous parents. Until the age of 5 months, development appeared normal. By the age of 5 months he manifested seizures, which initially had a focal character, predominantly affecting the upper limb with a duration of 1-2 minutes and a frequency of 2-3 times a day. Seizures gradually became generalized, encompassing generalized tonic-clonic seizures and focal seizures with secondary generalization. In line with the clinical features, the initial EEG showed bifrontal epileptiform discharges, which later progressed to generalized cortical slowing with intermittent asynchronous activity. Seizures were refractory to treatment with anti-epileptic drugs, including phenobarbital, carbamazepine, topiramate and levetiracetam.

He had severe intellectual disability and delayed motor development. Yet, by the age of 24 months he was able to walk and climb upstairs at 30 months of age. The attainment of social milestones and development of speech were severely delayed and he had no sphincter control. At later age, he started to display autistic features, self-mutilation, hyperphagia and ADHD. Sleep was disturbed. Neurological examination did not reveal hypotonia, spasticity or signs of movement disorders and brain MRI at the age of 6 years showed mild cerebellar vermis atrophy. His older brother, not available for further studies, also displayed intellectual disability and seizures (V2 in family 11, **Supplementary Fig. 1**), as well as three sons of two parental uncles (also currently not available for studies).

To identify the cause of the clinical features, a high-resolution karyotyping and Fragile X analyses were performed, which returned normal. This prompted the conduction of exome sequencing.

Family 12

Patient 12A (P12A), currently 6 years of age, was born at term to consanguineous parents (**Supplementary Fig. 1**) of Arabic descent after an uncomplicated pregnancy. At the age of 8 months, he first presented seizures. At first, he had seizures with a focal onset with impaired awareness (non-motor onset – behavior arrest) and later generalized tonic and tonic-clonic seizures on a daily basis, which were refractory to different (combined) treatment regimes. The first EEG at the age of 9 months was normal, but later recordings showed left parietal focal epileptic discharge (at 12 months), generalized and multifocal epileptic discharges on a slow background pattern from 20 months onward.

At present, he has severe intellectual disability and is non-verbal. Even though achievement of motor milestones was delayed, he is able to walk unsupported from the age of 2.5 years. He does not exhibit pronounced hypotonia or spasticity and has a normal gait pattern. He is diagnosed with autism spectrum disorder (ASD) and has significant sleep disturbances.

Brain MRI performed at the age of 5 years revealed mild cerebellar atrophy, with prevalent superior vermis involvement, and a short corpus callosum with an enlarged perivascular space in the splenial region (Fig. 1A).

P12A died at the age of 6 years from a status epilepticus and liver failure of unknown origin.

Patient 12B (P12B), the younger brother of P12A, is currently 4 years old and was also born at term after an uncomplicated pregnancy. By the age of 18 months, he first presented seizures, which typically entail behavioral arrest with chewing and impaired awareness (but still responsive, albeit slow), and focal clonic jerking (right-sided) on occasion. Seizures are typically short (10-20 second) and were initially present on a daily basis. Currently, he is seizure free whilst on carbamazepine. An EEG at the age of 18 months did not show any abnormalities, whereas EEG recordings at the age of 20 months showed focal epileptic discharge in the right temporal lobe.

He had no delay in motor development and currently has some verbal speech. He does not exhibit any abnormalities on neurological examination, but exhibits behavioral problems including hyperphagia and sleep disturbances. Brain MRI at the age of 3 years showed mild cerebellar atrophy, with prevalent superior vermis involvement (**Fig.1A**).

Chromosomal microarray and basic metabolic workup was performed on the older sibling and was normal. Considering consanguinity and suspicion of a shared diagnosis for these brothers, trio exome sequencing involving both brothers and their mother was pursued.

Family 13

Patient 13A (P13A) was born at term to consanguineous parents. Prenatal ultrasonography had revealed a flexed hand and club food. Shortly after birth she started to manifest refractory seizures, which began as focal seizures and later progressed to myoclonic attacks. Seizures were frequent and usually lasted <1 minute, with clustering and several episodes of status epilepticus have been documented. EEG examination showed multiform epileptiform discharges.

At last evaluation at the age of 0.8 years old, she had profound intellectual disability and did not attain early developmental milestones. She exhibited severe hypotonia and spasticity. Brain MRI showed global brain atrophy. She died at the age of 0.8 years due to aspiration pneumonia.

Her younger brother, patient 13B (P13B), also born at term, showed a similar phenotype (**Table 1**) and manifested early onset refractory seizures. He died at the age of about 1 month due to respiratory failure.

Given the clinical phenotype and poor prognostic outcome of both siblings, exome sequencing of both parents and a subsequent fetus was performed during prenatal diagnostics to identify any hereditary causes.

Family 14

Patient 14 (P14) was born at-term to non-consanguineous parents and showed contractual arachnodactyly and low-set ears. At day 2 after birth, she started to manifest refractory seizures with a multi-focal character. She experienced multiple status epilepticus and clusters of seizures. EEG examination at the age of 1 and 2 months showed multi-focal, paroxysmal fast activity with sharp waves as well as bursts with irregular activity with spikes and polyspikes resembling hypsarrhythmia, pronounced over the left temporal hemisphere. Despite a ketogenic diet and treatment with different combinations of anti-epileptic drugs (including *e.g.* phenobarbital, lacosamide, midazolam and levetiracetam), seizures remained refractory.

Until the age of 6 months, she showed severe global developmental delay and remained non-responsive to environmental cues and did not attain head control. Initial MRI at the age of 2 months was normal, but an MRI at the age of 5 months showed no progress of myelination. She died in palliative care at the age of 6 months.

Given the global developmental delay and refractory seizures, exome sequencing was performed on patient and both parents to identify putative genetic causes.

Family 15

Patient 15A (P15A), currently 16-years old, was born at-term to consanguineous parents, with a normal birth weight. At birth, she had dysmorphic features including prominent maxillae and lips, clinodactyly on fifth finger of the left arm. Starting from post-natal day 23, he started to exhibit tonic-clonic seizures on a daily basis, which lasted for several minutes, showed clustering and were refractory to anti-epileptic therapy. EEG examination showed diffusely slow and dysregulated background activity and multifocal epileptiform discharges. MRI examination showed cerebral atrophy.

By the age of 10 years, she showed severe global developmental delay, although she attained full head control by the age of 1 year and is now able to walk unsupported. Spastic features were present, but profound hypotonia was absent. She remained non-verbal and displayed autistic and several behavioral features, including irritability, self-mutilation, and stereotypic hand movements.

Patient 15B (P15B), the 8-years old sister of P15A, showed similar clinical features as her older sister, including intractable epilepsy and severe developmental delay. Another daughter was diagnosed with West syndrome and died at the age of 9 months.

Given the clinical phenotype in P15A and P15B, exome sequencing was performed on both patients and their parents to identify putative genetic causes.

Family 16:

Patient 16 (P16) was born at term to consanguineous parents (**Supplementary Fig. 1**) of Indian descent with normal antenatal and birth history. He first presented seizures at day 3 after birth. At first, he had tonic seizures generalized and was admitted in neonatal intensive care unit and was discharged on multiple AEDs. Despite these interventions, he had 2-3 episodes on a daily basis, lasting for minutes. At the age of 2 months, he developed myoclonic seizures and was started on valproate. Six status epilepticus have been documented. EEG examinations showed multifocal epileptiform dischrages. Seizures were difficult to control, despite the combined use of multiple antepileptics.

On examination, he had spasticity and profound intellectual disability. Verbal speech is absent. Brain MRI at the age of 6 months showed cerebral atrophy. He needed mechanical ventilation due to refractory status epilepticus with respiratory failure and died at the age of 26 months.

Genetic analyses

Exome variant data of the affected individuals were analyzed for rare and potentially deleterious autosomal or X-linked recessive variants. Homozygous or compound heterozygous variants in *ADAM22* were identified in all affected individuals with available genetic analyses (**Fig. 2**). P1 was homozygous for a missense variant c.1915C>A encoding the p.(His639Asn) substitution (variant nomenclature based on RefSeq ID NM_021723.3). This variant is present in a heterozygous state in two individuals of European/other ancestry in the gnomAD database (https://gnomad.broadinstitute.org/; last accessed on 06-06-2020), yielding an allele frequency of 7.31e-6. In addition, a c.[412dup] p.(Val138GlyfsTer49) variant in *NRXN1* was identified which did not segregate with the clinical phenotype as it was also present in the healthy mother. P2 was compound heterozygous for the same c.1915C>A; p.(His639Asn) variant on the paternal allele and a large deletion of 7q21.12(87,576,407-87,737,435), including the *ADAM22* locus was inherited from the healthy mother (identified through a SNP array). P3A was homozygous for a c.2077-2A>C variant, abolishing a branch-point nucleotide and the splice acceptor site of exon 24 with a predicted outcome involving exon 24 skipping (p.(Cys694LeufsTer7)). However, *in vitro* RNA studies indicated the activation of a cryptic splice acceptor site eight nucleotides downstream from the native splice site that immediately introduced a premature stop codon (r.2077_2084del, (p.(Val693*)) (**Supplementary Fig. 3A-C**). In absence of patient-derived brain samples, we were unable to pinpoint what mechanism predominantly occurs in the patient. In both cases, c.2077-2A>C variant transcripts containing premature stop codons are likely to undergo nonsense-mediated decay (NMD). Both parents were heterozygous for this variant. The c.2077-2A>C variant is not present in several large databases (**Supplementary Table 2**), although two other variants (c.2077-4T>C and c.2077-8G>A) affecting the same splice site have been identified in five and one heterozygous carriers, respectively. In addition, P3A was heterozygous for a c.3469G>A p.(Val1157Ile) variant in

MYH3 (NM_002470.3) and a c.1927G>A p.(Asp643Asn) variant in *CACNA1A* (NM_023035.2). Genetic analyses could not be performed in P3B, who deceased at young age, due to absence of genomic DNA. However, given the familial link to P3A (**Supplementary Fig. 1**), the great similarities in clinical presentation, and the presence of the same c.2077-2A>C variant in the parents (both healthy heterozygous carriers), P3B probably was homozygous for this variant. P4 was compound heterozygous for a c.1733C>T variant, resulting in a p.(Thr578Met) substitution and a c.2576+1G>C variant that, abolishes the splice donor site of exon 29 and results in aberrant splicing of two transcripts. The predominant transcript shows a skipping of exon 29 (r.2510_2576del) that causes a frameshift (p.(N838LfsTer35), while a minor fraction of transcripts indicate the activation of a cryptic splice donor site in intron 29 (r.2576_2576+1ins37; p.(E859DfsTer2)) (**Supplementary Fig. 3D-F**). Although this variant was absent across several large genomic databases, another variant at the same position (c.2576+1G>A) was present in a heterozygous state in one subject (**Supplementary Table 2**). In addition, P4 was heterozygous for a variant of unknown significance (VUS) in *DHCR7* and harbored two missense VUS in *UBE3A*. Since biological parents were not available for genetic testing, we were unable to perform a segregation analysis of family 4. P5 was homozygous for a c.1312C>A variant, resulting in a p.(Pro438Thr) substitution. This variant is not present in several large genomic databases. P6 was homozygous for a c.1343G>A variant, resulting in a p.(Gly448Asp) substitution and P7 was homozygous for a c.2686C>T; p.(Arg896*) variant. Both variants were absent across several large genomic databases (**Supplementary Table 2**). Both, P8 and P9, though unrelated, were homozygous for the c.1733C>T; p.(Thr578Met) variant, which had also been identified in P4. P10 has been previously reported and compound heterozygous for the c.1202G.A; p.(Cys401Tyr) and c.2396delG; p.(Ser799IlefsTer96) variants (2). P11, recently reported by Maddirevula et al. (2019), as well as P12A and P12B were homozygous for the same c.2686C>T; p.(Arg896*) as identified in P7. Although DNA of P13A

and P13B was not available for genetic testing, a homozygous c.1744A>G; p.(Asn582Asp) variant was identified during prenatal diagnostics of a subsequent fetus. Both parents were found heterozygous for this variant, which is absent across several large genomic databases (**Supplementary Table 2**). P14 was compound heterozygous for c.247-21179_390+8515del variant, resulting a deletion of exon 3 and 4 (p.(Leu83_Lys130del)) and a c.1421G>T; p.(Cys474Phe). Both parents were found heterozygous for this variant. P15A and B were homozygous for the same c.1312C>A; p.(Pro438Thr) variant as identified in P5. Patient 16 had a novel homozygous c.2433G>A; p(Trp811Ter) in exon 28, which was absent across various databases. Both parents were found heterozygous for this variant.

All identified (missense) variants affect highly conserved residues (**Fig. 2A**) and were predicted to be deleterious by commonly used *in silico* prediction tools (**Supplementary Table 2**).

Post-mortem examination of ADAM22 deficient human brain tissue

Brain tissue of P10 was available for post-mortem examination. The brain weight was only 482g, which amounts to ~35% of reported reference ranges in male subjects of similar age (reported mean brain weight 1,330-1,470 g, summarized in (6)). Extreme cortical atrophy was observed, increasing rostro-caudally in the neocortex. The hippocampi were very small, but neuronal cells were better preserved than in the cortical regions. Atrophy of the white matter and brain stem was interpreted as secondary to the cortical degeneration. The cerebellum showed less pronounced changes. The deep gray matter was quite preserved in striatum, but the thalami were very atrophic and gliotic. The neocortical atrophy, especially frontally, was quite total at the sulci, with some neurons preserved at the gyral regions. This pattern of

neuropathology (**Fig 1B-E)** has some resemblance with Alpers disease, although the large number of corpora amylacea is not common in Alpers.

Author contributions

MvdK, YvI, YH-H, RM, HH, CP-S, DJK, AP, AEL, ISP, VYV, S.S., P.L. F.S.A., S.M., H.M. performed the clinical assessments, or provided clinical information of the affected individuals. C.A.L.R. performed the genetic analysis in P1 and P2. M.M. performed the genetic analysis in P10, S.W. and T.B.H. of P14, and I.S.P of P7. EEG recordings were centrally analyzed by P.S. and M.S. (re-)evaluated the available MRIs. Y.F., Y.M., Y.H., and M.F. performed the functional characterization of ADAM22 variants. A.R. and B.V. performed the in vitro splice assay. MvdK, YH-H, M.F., Y.F., B.V., R.M., and H.H. wrote the manuscript. All authors critically reviewed the manuscript.

References

1. Tompson SW, Young TL. Assaying the Effects of Splice Site Variants by Exon Trapping in a Mammalian Cell Line. Bio Protoc. 2017;7(10).
2. Muona M, Fukata Y, Anttonen AK, Laari A, Palotie A, Pihko H, et al. Dysfunctional ADAM22 implicated in progressive encephalopathy with cortical atrophy and epilepsy. Neurol Genet. 2016;2(1):e46.
3. Fukata Y, Adesnik H, Iwanaga T, Bredt DS, Nicoll RA, Fukata M. Epilepsy-related ligand/receptor complex LGI1 and ADAM22 regulate synaptic transmission. Science. 2006;313(5794):1792-5.
4. Yamagata A, Miyazaki Y, Yokoi N, Shigematsu H, Sato Y, Goto-Ito S, et al. Structural basis of epilepsy-related ligand-receptor complex LGI1-ADAM22. Nat Commun. 2018;9(1):1546.
5. Maddirevula S, Alzahrani F, Al-Owain M, Al Muhaizea MA, Kayyali HR, AlHashem A, et al. Autozygome and high throughput confirmation of disease genes candidacy. Genet Med. 2019;21(3):736-42.
6. Govender S, Lazarus L, De Gama BZ, Satyapal KS. Post-mortem brain weight reference range for a select south african population. Int J Morphol. 2018;36(3):915-20.
7. van der Sluijs PJ, Aten E, Barge-Schaapveld D, Bijlsma EK, Bokenkamp-Gramann R, Donker Kaat L, et al. Putting genome-wide sequencing in neonates into perspective. Genet Med. 2019;21(5):1074-82.
8. Bauer P, Kandaswamy KK, Weiss MER, Paknia O, Werber M, Bertoli-Avella AM, et al. Development of an evidence-based algorithm that optimizes sensitivity and specificity in ES-based diagnostics of a clinically heterogeneous patient population. Genet Med. 2019;21(1):53-61.

9. Efthymiou S, Dutra-Clarke M, Maroofian R, Kaiyrzhanov R, Scala M, Reza Alvi J, et al. Expanding the phenotype of PIGS-associated early onset epileptic developmental encephalopathy. Epilepsia. 2021;62(2):e35-e41.
10. Park J, Colombo R, Schaferhoff K, Janiri L, Grimmel M, Sturm M, et al. Novel HIVEP2 Variants in Patients with Intellectual Disability. Mol Syndromol. 2019;10(4):195-201.
11. Fukata Y, Chen X, Chiken S, Hirano Y, Yamagata A, Inahashi H, et al. LGI1-ADAM22-MAGUK configures transsynaptic nanoalignment for synaptic transmission and epilepsy prevention. Proc Natl Acad Sci U S A. 2021;118(3).

Supplementary Tables

Supplementary Table 1 Clinical characteristics of 20 out of 21 affected individuals with pathogenic variants in *ADAM22* and available clinical data, including an overview from literature

	**P1**	**P2**	**P3A**	**P3B**	**P4**	**P5**	**P6**	**P7**	**P8**	**P9**	**P10** (2)	**P11** (5)†	**P12A**	**P12B**	**P13A**	**P13B**	**P14**	**P15A**	**P15B**	**P16**
Age in years *	12	7	1.3*	0.5*	7	1.7	19	3	4	0.6*	28*	19	6*	4	0.8*	0.1*	0.5*	16	8	2.2*
Alive (age at death in years; cause of death)	Yes	Yes	No (1.3; cardio respiratory failure)	No (0.5; respiratory failure)	Yes	Yes	Yes	Yes	Yes	No (0.6; n.a.)	No (28; pneumonia)	Unknown	No (6; status epilepticus, liver failure)	Yes	No (0.8; aspiration pneumonia)	No (0.1; respiratory failure)	No (0.5; palliative care)	Yes	Yes	Yes (2.2; respiratory failure)
Gender	F	F	F	F	M	M	F	M	M	M	F	M	M	M	F	M	F	F	F	M
Ethnicity	Dutch	Dutch	Israeli Druze	Israeli Druze	American-European	Persian	Brazilian	Armenian/ Yezidi	Egyptian	Persian	Finnish	Arab	Iranian Arab	Iranian Arab	Arab	Arab	German	Turkish	Turkish	Indian
**Genetics**																				
DNA	c.1915C>A	c.1915C>A/ del 7q21.12 (87,576,407-87,737,435)†	c.2077-2A>C	*c.2077-2A>C*	c.1733C>T/ c.2576+1G>C	c.1312C>A	c.1343G>A	c.2686C>T	c.1733C>T	c.1733C>T	c.1202G>A /c.2396delG	c.2686C>T	c.2686C>T	c.2686C>T	c.1744A>G	c.1744A>G	c.247-21179_390+8515del;c.1421G>T	c.1312C>A	c.1312C>A	c.2433G>A
Protein	p.(His639Asn)	p.(His639Asn) / -	p.(Cys694Leufs Ter7)	*p.(Cys694Leufs Ter7)*	p.(Thr578Met) / p.(Glu859AspfsTer2)	p.(Pro438Thr)	p.(Gly448Asp)	p.(Arg896*)	p.(Thr578Met)	p.(Thr578Met)	p.(Cys401Tyr) / p.(Ser799IlefsTer96)	p.(Arg896*)	p.(Arg896*)	p.(Arg896*)	p.(Asn582Asp)	p.(Asn582Asp)	p.(Leu83_ Lys130del)/ p.(Cys474Phe)	p.(Pro438Thr)	p.(Pro438Thr)	p.(Trp811Ter)
Zygosity	hom	comp. het.	Hom	hom	comp. het.	hom	hom	hom	hom	hom	comp. het.	hom	hom	hom	hom	hom	comp. het.	hom	hom	hom
Sequence method	WES (trio) + SNP array (7)	WES (trio) +SNP array (7)	WES (8)	Sanger of parents ⁋	WES (8)	WES (8)	WES ‡	WES ‡	WES (8)	WES (9)	WES (2)	WES (5)	WES (mother and sibling) (9)	WES (mother and sibling) (9)	WES (TRIO) (8)	WES (TRIO) (8)	WES + Sanger of parents (10) ‡	WES (parents and sibling)	WES (parents and sibling)	WES + Sanger of parents
**Pregnancy and birth**																				
Gestational age (w)	40+2	42+1	39+1	38+2	n.a.	40	37	40	36	n.a.	At term	At term	40	40	38	38	40+6	38	38	38
Birth weight in gr (SD)	3275 (-0.4)	3930 (+0.5)	2620 (-1.4)	1847 (-2.8)	2721 (n.a.)	3850 (+0.2)	2640 (-0.4)	3150 (-0.8)	3200	n.a.	3700 (+0.4)	3500 (-0.4)	3310 (-0.1)	n.a.	3000 (-0.1)	3000 (-0.1)	3240 (-0.7)	3500	3000	3000
HC in cm (SD)	n.a.	n.a.	33.5 (-0.3)	n.a.	n.a.	35.0 (+0.4)	32.0 (-0.8)	n.a.	n.a.	n.a.	33.5 (-0.3)	n.a.	n.a.	n.a.	33.5 (-0.8)	34 (-0.4)	36.5 (+1.0)	n.a.	n.a.	n.a
**Seizures**																				
Age of onset in m	7.0	2 .0	0.1	0.1	0.1	1.3	2.5	18.0	6.0	3.0	3.0	5.0	8.0	18.0	Birth	Birth	2 days	0.8	2	3 days
Type	Focal non-motor (temporal) seizures, myoclonic, tonic-clonic	Focal > multifocal, spasms	myoclonic, tonic	Tonic-clonic	Focal > multifocal	Focal motor (right side), tonic clonic	Focal motor	Focal > multifocal	Tonic-clonic seizures Myoclonic	Tonic	Focal motor	Focal motor	Focal non-motor > generalized tonic clonic	Multi-focal (motor and non-motor)	Focal > myoclonic	Focal > myoclonic	Multi-focal (temporal left side accentuated)	Tonic-clonic	İnfantile spasms, tonic-clonic, myoclonic	Tonic seizures, Myoclonic seizures
Status epilepticus (age)	No	Yes (5 m)	Yes (unknown)	Unknown	Yes (5 m)	Yes (n.a.)	Yes (<12m)	No	No	Yes (unknown)	No	Yes (unknown)	No	No	Yes	Yes	Yes (from age 2 days)	Yes (2 m)	Yes (3 m)	Yes
Daily episodes (n)	Yes	Yes (>10)	Yes	Yes	Yes (10-40)	Yes	No	Yes (>10)	n.a.	n.a.	n.a.	Yes	Yes	Yes	Yes	Yes	Yes	Yes	Yes	Yes
Duration	<1 min	0.5-3 min	<1 min	variable	10-15 sec	3 min - 1 h	< 1 min	< 3 min	n.a.	n.a.	n.a.	1-2 min	0.5-1.5 min	10-20 sec	<1 min	<1 min	n.a.	1-3 min	3-4 min	3-4 min
Clustering	Yes	No	Yes	Yes	Yes	no	n.a.	Yes	n.a.	n.a.	n.a.	n.a.	No	No	Yes	Yes	Yes	Yes	Yes	Yes
EEG abnormalities (age)	Diffusely low-voltage background activity and rare, small sharp waves central areas (5 y); diffuse slowed and dysregulated background activity and occasional theta rhythms frontal and central areas of both hemispheres (10 y)	Hypsarrhythmia	Irregular, slow wave and sharp wave complexes over the posterior left regions within a diffusely disorganized and slowed background activity (3 m)	Paroxysmal discharges with epileptiform template (periodic lateralized epileptiform discharges PLEDs) - slow peaked waves mainly over the right hemisphere, on the background of multifocal epileptic activity.	n.a.	Multifocal paroxysmal activity over the central and posterior regions of both hemispheres (at onset)	Diffuse slowed and dysregulated background activity and occasional slowing over the (L>R) central and temporal regions (18 y)	Multifocal (L>R) waves and spikes/sharp waves; hypsarrhythmia	Hypsarrhythmia	Multifocal sharp waves over the central regions within a diffusely disorganized activity	Secondarily generalized left spikes; slow background activity	Bifrontal epileptiform discharges > Diffusely slowed background activity	Left parietal focal epileptic discharge (12m) > diffuse slowing, generalized and multifocal epileptic discharges (17m) > Generalized epileptic discharge (>20m)	Focal epileptic discharge temp lobe (20m)	Multiform epileptiform discharges	Multiform epileptiform discharges	Multi-focal paroxysmal fast activity with sharp waves as well as bursts with irregular activity with spikes and polyspikes resembling hypsarrhythmia, pronounced over the left temporal hemisphere (1 and 2m)	Diffusely slow and dysregulated background activity and multifocal epileptiform discharges	Hypsarrhythmia	Multiform epileptiform discharges
Refractory	Yes	Yes	Yes	Yes	Yes	Yes	Yes	Yes	Yes	Yes	Yes	Yes	Yes	No	Yes	Yes	Yes	Yes	Yes	Yes
**Growth and development**																				
Weight in SDs (age)	+0.7 (6y)	-0.6 (7y6m)	n.a.	+2.9 (7y)	-0.3 (1y2m)	-1.8 (18y)	-1.8 (18y)	+2.58 (3y)	n.a.	n.a.	n.a.	n.a.	-1.1 (5y)	+1.9 (3.3y)	-1.5 (0.8 y)	-1.2 (0.1 y)	n.a.	-1.3 (10 y)	-0.7 (2 y)	-2.48
Height in SDs (age in yrs)	-1.7 (5y7m)	-1.4 (5y6m)	n.a.	+0.9 (7y)	-2.4 (1y2m)	-2.5 (18y)	-2.5 (18y)	+0,69 (3y)	n.a.	n.a.	n.a.	n.a.	-2.1 (4.4y)	-0.3 (3.3y)	+0.5 (0.8 y)	+0.5 (0.1 y)	n.a.	n.a.	-0.7 (2 y)	-2.44
HC in SDs (age in yrs)	-1.4 (5y7m)	-1.4 (5y6m)	n.a.	+1.8 (7y)	-0.3 (1y2m)	-2.0 (13y11m)	-2.0 (14y)	-0.1 (3y)	n.a.	n.a.	-1.2 (12y)	-1.2 (12y)	-0.2 (4.4y)	+1.6 (3.3y)	-1.1 (0.8 y)	-1.6 (0.1 y)	n.a.	-2.0 (10 y)	-4.2 (2 y)	-2.55

	Intellectual disability	Severe	Severe	Profound	Profound	Severe	Severe	Moderate-severe	Moderate-severe	Yes	Yes	Profound	Severe	Severe	n.a.	Severe	Severe	Non-responsive	Severe	Severe	Profound
	Delayed motor milestones	Severe	Severe	Profound	Profound	Severe	Severe	Mild	No	Profound	Profound	Profound	Mild	Yes	No	Yes	Yes	Severe	Severe	Severe	Profound
	Head control	Yes	Yes	n.a.	n.a.	Yes	n.a.	Yes	Yes	No	No	No	Yes	Yes	Yes	No	No	No	Yes (1 y)	Yes (1.5y)	No
	Sits unsupported	Yes	Yes	n.a.	n.a.	No	n.a.	Yes (~12 m)	Yes	No	No	No	Yes	Yes	Yes	N.a.	N.a.	No	Yes	Yes	No
	Walks unsupported	Yes	No	n.a.	n.a.	No	n.a.	Yes (20 m)	Yes (12 m)	No	No	No	Yes (2.0 y)	Yes (2.5 y)	Yes	No	No	No	Yes	No	No
	Verbal speech	No	No	No	No	No	No	No	No	No	N.a.	No	No	No	Yes (but delayed)	No	No	No	No	No	No
	Autistic features	Yes	No	No	No	No	No	Yes	No	No	N.a.	No	Yes	Yes	No	No	No	N.a.	Yes	Yes	No
	Behavioral problems	Aggression, self-mutilation	No	N.a.	N.a.	Aggression	N.a.	No	ADHD	Irritability	N.a.	No	ADHD, self-mutilation	Self-stimulation, head banging	Hyperphagia	No	No	N.a.	Yes (irritability, self-mutilation, stereotypic hand movements)	Yes (irritability, self-mutilation, stereotypic hand movements)	No
**Clinical examination**																					
	Hypotonia	No	Yes	Yes	Yes	Yes	Yes	Yes	No	Yes	Yes	Yes	No	No	No	Yes	Yes	No	No	Yes	No
	Spasticity	No	Yes	No	No	Yes	No	No	No	No	N.a.	Yes	No	No	No	Yes, pre-natal	Yes	No	Yes	Yes	Yes
	Extrapyramidal signs	No	Wringing of hands	No	No	No	No	Impaired coordination	No	Abnormal choreic movements	N.a.	No	No	No	No	No	No	No	No	No	No
	Brain MRI (age)	Mild cerebral atrophy with reduced WM volume (8 m)§	Normal (2.5 m); Mild frontal atrophy with reduced WM volume, WM signal alterations enlarged PVS in the CC (2.5y). Normal MRS (2.5 y) §	Normal (3 m)	Moderate cerebral atrophy with reduced WM volume, normal MRS (4 m)	WM signal alterations, CC hypoplasia, posterior thalami signal changes, cerebellar atrophy (3y) §	Mild cerebral atrophy with reduced WM volume and CC thinning (4 m) §	Left mesial temporal sclerosis and focal cortical dysplasia (17.5 y) §	Mild frontal atrophy with reduced WM volume (2y) §	Mild cerebral atrophy (1 y)	Moderate cerebral and cerebellar atrophy, reduced WM volume, DM, CC thinning (3m and 4m) §	Severe cerebral atrophy with reduced WM volume (11 y)	Mild cerebellar vermis atrophy (6 y) §	Mild cerebellar atrophy, with prevalent vermis involvement, short CC with enlarged PVS (5y)§	Mild cerebellar atrophy, with prevalent vermis involvement (3y)§	Cerebral atrophy (n.a.)	Cerebral atrophy (n.a.)	Initial MRI normal (2m); no progress of myelination (5m)	Cerebral atrophy (n.a.)	Cerebral atrophy (n.a.)	Cerebral atrophy

* in case the proband is deceased, the age of death is indicated

† Maddirevula et al (2019) used the RefSeq ID NM_016351.4 for the *ADAM22* gene (one of the short spliced forms of *ADAM22*) to denote the variant (c.2578C>T; (p.Arg860*)) in the original paper, instead of the RefSeq ID NM_021723.3 (a long spliced form of ADAM22) used in this manuscript.

§ Individuals in whom original brain imaging was available for central reassessment.

⁋ Since no DNA was available of P3B due to death at young age, the presence of a bi-allelic pathogenic variant in P3B was inferred from the identification of pathogenic variants on one allele in both parents and based on the strong similarities in clinical phenotype compared to P3A within the same family (Supplementary Fig. 1).

‡ Sequencing methods of P6 and P7 have not been previously published. Briefly, for P6 whole-exome sequencing was performed using Sureselect Human All Exon v7 (Agilent) and HiSeq platform (Illumina), methods of P7 are available through https://doi.org/10.1101/2021.02.02.429394. Sequencing methods of P14 have been described previously[32], with minor modifications (kit version V7, instrument NovaSeq 6000; paired-end reads: 104 bp).

Corresponding pedigrees are available in Supplemental Fig 1 and detailed case descriptions are in the Supplemental Result section.

Abbreviations: CC, corpus callosum; comp. het., compound heterozygous; CSF, cerebrospinal fluid; DM, delayed myelination; HC, head circumference; SD, standard deviation; hom, homozygous; MRI, magnetic resonance imaging; MRS, magnetic resonance spectroscopy; PVS, perivascular spaces; WM, white matter; n.a., not available/applicable.

Supplementary Table 2 Genetic findings in the 20 affected individuals with pathogenic/likely pathogenic missense, nonsense or frameshift and splicing variants and three non-pathogenic missense variants in *ADAM22*

	**Patient ID**	**P1-2**	**P3A-B**	**P4,8,9**	**P4**	**P5, 15A-B**	**P6**	**P7, 11, 12A-B (5)**	**P10 (2)**	**P10 (2)**	**P13A-B**	**P14**	**P16**	**Nonpathogenic variant 1**	**Nonpathogenic variant 2**	**Nonpathogenic variant 3**
**Variant Annotation**	gDNA change (GRCh38/hg38)	chr7:88,163,019 (C>A)	chr7:88,165,830 (A>C)	chr7:88,153,272 (C>T)	chr7:88,182,025 (G>C)	chr7:88,143,117 (C>A)	chr7:88,145,147 (G>A)	chr7:88,193,225 (C>T)	chr7:88,136,013 (G>A)	chr7:88,179,030 (delG)	chr7:88,153,283 (A>G)	chr7:88,145,443 (G>T)	Chr7:87,810,844 (G>A)	chr7:87,935,103 (C>T)	chr7:88,128,617 (C>T)	chr7:88,193,219 G>A
	cDNA change (NM_021723.5)	c.1915C>A	c.2077-2A>C	c.1733C>T	c.2576+1G>C	c.1312C>A	c.1343G>A	c.2686C>T	c.1202G>A	c.2396delG	c.1744A>G	c.1421G>T	c.2433G>A	c.163C>T	c.694C>T	c.2680G>A
	Protein change	p.(His639Asn)	p.(Cys694Leufs*7)	p.(Thr578Met)	p.(Glu859Aspfs*2)	p.(Pro438Thr)	p.(Gly448Asp)	p.(Arg896*)	p.(Cys401Tyr)	p.(Ser799Ilefs*96)	p.(Asn582Asp)	p.(Cys474Phe)	p.(Try811Ter)	p.(Leu55Phe)	p.(Arg232Cys)	p.(Val894Met)
	Zygosity	Hom / CompHet	Hom	CompHet / Hom	CompHet	Hom	Hom	Hom	CompHet	CompHet	Hom	CompHet	Hom	-	-	-
	dbSNP ID	rs374179429	NA	NA	NA	rs1586143253	NA	rs1262642807	rs747259064	rs1554519462	-	-	-	rs372922125	rs376280562	rs182800008
	Variant found in family	1/2	3	4/8/9	4/15	5	6	7/11/12	10	10	13	14	-	-	-	-
**Allele frequencies**	gnomAD v3.1.2	3 hets	Absent	1 het	Absent	Absent	Absent	Absent	1 het	Absent	Absent	Absent	Absent	24 hets, 1 hom	18 hets	198 hets, 1 hom
	gnomAD v2.1.1	2 hets	Absent	Absent	Absent	Absent	Absent	Absent	2 hets	Absent	Absent	Absent	Absent	226 hets, 1 hom	67 hets, 1 hom	184 hets, 1 hom
	Highest frequency in Ensembl browser	0.00000796400% (TOPMed)	-	-	-	0% (NCBI ALFA)	-	0.002096% (gnomAD v.3.0)	0.000008056% (gnomAD v.2.1.1)	Absent	-	-	-	0.001% (gnomAD v.2.1.1)	0.000273% (gnomAD v.2.1.1)	0.002% (1000 Genomes)
	Iranome	Absent	Absent	Absent	Absent	Absent	Absent	Absent	Absent	Absent	Absent	Absent	-	Absent	1 het	Absent
	GME Variome	Absent	Absent	Absent	Absent	Absent	Absent	Absent	Absent	Absent	Absent	Absent	-	Absent	Absent	6 hets
	UK Biobank, 537.492 Alleles	Absent	Absent	2 hets	Absent	Absent	Absent	1 het	Absent	Absent	Absent	Absent	-	5 hets	10 hets	130 hets
	Centogene, 82.000 Alleles	1 het	Absent	2 hets	Absent	2 hets	Absent	Absent	1 het	Absent	2 hets	Absent	-	-	-	-
	TOPMed, 125.568 Alleles	1 het	Absent	Absent	Absent	Absent	Absent	2 hets	Absent	Absent	Absent	Absent	-	23 hets	13 hets	337 hets
	QSG 50.000 Alleles	Absent	Absent	Absent	Absent	2 hets	Absent	Absent	Absent	Absent	Absent	Absent	-	35 hets, 1 hom	29 hets, 2 homs	61 hets, 3 homs
***In silico* predictions**	GERP	5.70	5.44	5.45	5.48	5.75	5.88	5.72	4.98	5.53	5.55	5.83		4.94	5.10	5.72
	CADD	25.5	34.0	25.4	32.0	25.4	28.1	44.0	30.0	NA	26.9	28.6	43.0	26.4	31.0	25.0
	Polyphen-2	Probably damaging (1.000)	NA	Probably damaging (0.998)	NA	Probably damaging (1.000)	Probably damaging (1.000)	NA	Probably damaging (1.000)	NA	Probably damaging (1.000)	Probably damaging (1.000)	NA	Probably damaging (1.000)	Probably damaging (0.915)	Benign (0.309)
	SIFT	Damaging (0.005)	NA	Damaging (0.001)	NA	Damaging (0.000)	Damaging (0.001)	NA	Damaging (0.000)	NA	Damaging (0.007)	Damaging (0.001)	NA	Damaging (0.000)	Damaging (0.000)	Damaging (0.011)
	Provean	Damaging (-5.83)	NA	Damaging (-5.25)	NA	Damaging (-7.73)	Damaging (-6.53)	NA	Damaging (-10.62)	NA	Damaging (-4.26)	Damaging (-9.89)	NA	Damaging (-2.86)	Damaging (-5.72)	Neutral (-0.63)
	MutationTaster	DC (1.0000)	DC (1.0000)	DC (1.0000)	DC (1.0000)	DC (1.0000)	DC (1.0000)	DC (1.0000)	DC (1.0000)	DC (1.0000)	DC (1.0000)	DC (1.0000)	DC (1.0000)	DC (0.9894)	DC (1.0000)	P (0.9958)
**ACMG criteria and classification**	Overall classification	Likely pathogenic (PS3, PM2, PP1, PP3, PP4)	Pathogenic (PVS1, PM2, PP1, PP3, PP4)	Pathogenic (PS3, PM2, PM3, PP1, PP3, PP4)	Pathogenic (PVS1, PS3, PM2, PP3, PP4)	Likely pathogenic (PS3, PM2, PP3, PP4)	Likely pathogenic (PS3, PM2, PP3, PP4)	Pathogenic (PVS1, PM2, PP1, PP4, PP5)	Likely pathogenic (PM2, PM3, PP3, PP4, PP5)	Pathogenic (PVS1, PS3, PM2, PP3, PP4)	Likely pathogenic (PS3, PM2, PP1, PP3, PP4)	Likely pathogenic (PS3, PM2, PM3, PP3, PP4)	Pathogenic (PVS1, PM1, PM2, PP3)	Benign (BS2, BS3)	Benign (BS2, BS3)	Benign (BS2, BS3, BP4, BP6)

Abbreviations: ACMG, American College of Medical Genetics and Genomics; Het, heterozygous, CompHet, Compound heterozygous; DC, Disease-causing; Hom, Homozygous; NA, Not applicable; P, Polymorphism; PM, Pathogenic moderate; PP, Pathogenic supporting; PVS, Pathogenic very strong.

Supplementary Table 3. Summary of functional defects in ADAM22 variants.
Primary defects of individual ADAM22 variants are indicated in [**bold**]. LOF, loss of function.

ADAM22 variants	Protein maturation	Total protein expression	Cell-surface expression	LGI1-binding	PSD-95-binding	Mode of action	Ref
Wild-Type	+	+	+	+	+		
Leu83_Lys130del	**[–]**	reduced	–	–	reduced	LOF	
Cys401Tyr	+	+	+	**[reduced]**	+	LOF	(2)
Pro438Thr	**[–]**	reduced	–	–	reduced	LOF	
Gly448Asp	**[–]**	reduced	–	–	reduced	LOF	
Cys474Phe	**[–]**	reduced	reduced	–	reduced	LOF	
Thr578Met	**[reduced]**	reduced	reduced	reduced	reduced	LOF	
Asn582Asp	**[reduced]**	reduced	+	reduced	reduced	LOF	
His639Asn	**[reduced]**	reduced	reduced	reduced	reduced	LOF	
Cys694Luefs*7	+	**[reduced]**	–	–	–	LOF	
Ser799Ilefs*96	**[–]**	reduced	–	–	–	LOF	(2)
Trp811* (#)	+	**[reduced]**	–	–	–	LOF	
Glu859Aspfs*2	+	+	+	+	**[–]**	LOF	
Arg896*	+	+	+	+	**[–]**	LOF	(11)
†Leu55Phe	+	+	+	+	+	-	
†Arg232Cys	+	+	+	+	+	-	
†Val894Met	+	+	+	+	+	-	

Primary defects of individual ADAM22 variants are indicated in [**bold**]. LOF, loss of function. #, not tested but expected.

†Homozygous variants from the gnomAD database which contains sequencing data from presumably healthy individuals.

Supplementary Table 4 Primer sequences for generating cDNA variants

**Variant**	**Primer sequence (5’-3’ direction)**
L83_K130del	GCGACCTCGGTGGCCCGCAGGGAGGAGAGCACTGTTACTA
P438T	GTGCCTGCCTTTTCAACAAAACTTCTAAGCTTCTTGATCCT
G448D	TCTTGATCCTCCTGAGTGTGACAATGGCTTCATTGAAACTG
C474F	CCTTGAAGGAGCAGAGTGTTTTAAGAAATGCACCTTGACTC
T578M	GAAACTGAATATTGAAGGGATGGAGAAGGGTAACTGTGGGA
N582D	TTGAAGGGACGGAGAAGGGTGACTGTGGGAAAGACAAAGAC
H639N	CATTAAACTGCAGTGGTGGGAATGTTAAGCTTGAAGAAGAT
C694LfsTer7	GCACTATTTGCTCAGGAAATGGAGTGTTGCTGGCACCAATATCATAA
E859DfsTer2	TTAGTCAGTTGAATTAGACCGAGGTCT
L55F	AGAGCATCGTGCCACTGCGCTTCATCTACCGCTCGGGCGGC
R232C	GGCAGCTTCGTCGATATCCTTGTAATGTAGAAGAAGAAACC
V894M	TTAAATGGATGTCTCCCATAGCCTGGCACTTTGTCGGTTCATTTTCTTGTCCTCATCAGG

The positions of the introduced variants are underlined.

Supplementary Figure legends

Supplementary Fig. 1. Pedigrees of all reported families.

†P2 is compound heterozygous for a large deletion of 7q21.12(87,576,407-87,737,435), including the ADAM22 locus, on the maternal allele.

Supplementary Fig. 2. Neuroimaging features

Neuroimaging features associated with ADAM22 variants, including cerebral atrophy with enlargement of the CSF spaces (arrows) and lateral ventricles (asterisks), cerebellar atrophy with prevalent vermian involvement (empty arrows), and corpus callosum hypoplasia/thinning (arrowheads). Note the rapid evolution of cerebral atrophy with delayed myelination in P9 scanned at 3 (P9') and 4 months of age (P9''). In P4, there is additional diffuse hyperintensity of the supratentorial white matter with bilateral pulvinar involvement (dotted arrows) on FLAIR images. In P6, there is left hippocampal sclerosis (dotted arrow) associated with T2 hyperintensity of the ipsilateral anterior temporal lobe white matter (thick arrow). In P12A there is a short CC with an enlarged perivascular space in the splenial region (dotted circles).

Supplementary Fig. 3. *In vitro* RNA analysis of the *ADAM22* c.2077-2A>C and c.2576+1G>C splice variants.

(**A**) Gel electrophoresis of the RT-PCR wild-type, c.2077-2A>C variant, and empty pSPL3 vector amplicons. Transfection negative and PCR negative controls performed as expected.

(**B**) *In silico* splice predictions of the wild-type (top, red A) and c.2077-2A>C (bottom, red C). The cryptic splice acceptor site that is activated due to the variant is underlined and marked with a red arrow and validated with the in vitro splice assay.

(**C**) Vector construct of the *in vitro* splice assay. Amplicons containing either the c.2077-2A>C variant causing aberrant splicing (upper splice profile) or wild-type (lower splice profile) were

inserted between exons A and B of the pSPL3 vector. A schematic of the wild-type c.2077-2 (left sequencing panel), homozygous c.2077-2A>C variant (middle sequencing panel) and empty vector control (right sequencing panel) with exon-exon junction sequences are illustrated.

(**D**) Gel electrophoresis of the RT-PCR wild-type, c.2576+1G>C variant, and empty pSPL3 vector amplicons. Transfection negative and PCR negative controls performed as expected.

(**E**) *In silico* splice predictions of the wild-type (top, red G) and 2576+1G>C (bottom, red C). The cryptic splice donor site that is activated due to the variant is underlined and marked with a red arrow and validated with the in vitro splice assay.

(**F**) Vector construct of the *in vitro* splice assay. Amplicons containing either the c.2576+1G>C variant causing aberrant splicing (upper splice profile) or wild-type (lower splice profile) were inserted between exons A and B of the pSPL3 vector. Illustrated cDNA amplicons are shown from left to right: A schematic of the wild-type c.2576+1, homozygous c.2576+1G>C variant with exon 29 skipped, homozygous c.2576+1G>C variant with an activated cryptic donor site, extending the transcript by 37 bp, and the empty vector control (right sequencing panel).

Supplementary Fig. 4. Functional evaluation of suspected pathogenic variants in patients identified during preparation of the manuscript.

(**A**) ADAM22 variants (C474F, N582D and putative L83_K130del) were expressed in COS7 cells, and cell-surface expressed ADAM22 (magenta) was live-labeled by an antibody against the extracellular domain of ADAM22.

(**B–D**) Indicated ADAM22 variants were co-expressed with LGI1-FLAG (B and C) or PSD-95-FLAG (D) in COS7 cells, and their interactions were tested as in Fig. 3. ADAM22 C474F and L83_K130del variants were mostly retained as immature forms and hardly bound to LGI1.

The maturation of ADAM22 N582D protein was reduced (C and D, input), and its binding to LGI1 and PSD-95 was greatly reduced. Arrows and Arrowheads indicate the positions of immature and mature forms of ADAM22, respectively. The data shown is representative of two experiments. Bars, 20 μm (A, B).

(**E**) Close-up view of Cys474 of ADAM22. The C474F variant impairs the disulfide bond formation between C458 and C474, which supports the Ca^{2+} coordination. It is speculated that the C474F variant destabilizes the disintegrin domain of ADAM22.

Supplementary Fig. 5. Functional analysis of three non-pathogenic variants in ADAM22.

(**A-C**) Three ADAM22 variants observed in gnomAD database (L55F, R232C and V894M) were co-expressed with LGI1-FLAG (A and B) or PSD-95-FLAG (C) in COS7 cells, and their interactions were tested as in Fig. 3. Three ADAM22 variants (indicated in magenta letters) bound to LGI1 (A, B) and PSD-95 (C) as wild-type ADAM22. T578M and E859DfsTer2 represent pathogenic variants as tested in Fig. 3. An arrow and an arrowhead indicate the positions of immature and mature forms of full-length ADAM22, respectively. IP, immunoprecipitation. Bar, 20 μm.

Supplementary Fig. 6. Full western blots presented in the manuscript

All western blots that are presented in the indicated figures of the manuscript are displayed in full.

Supplementary Fig. 1

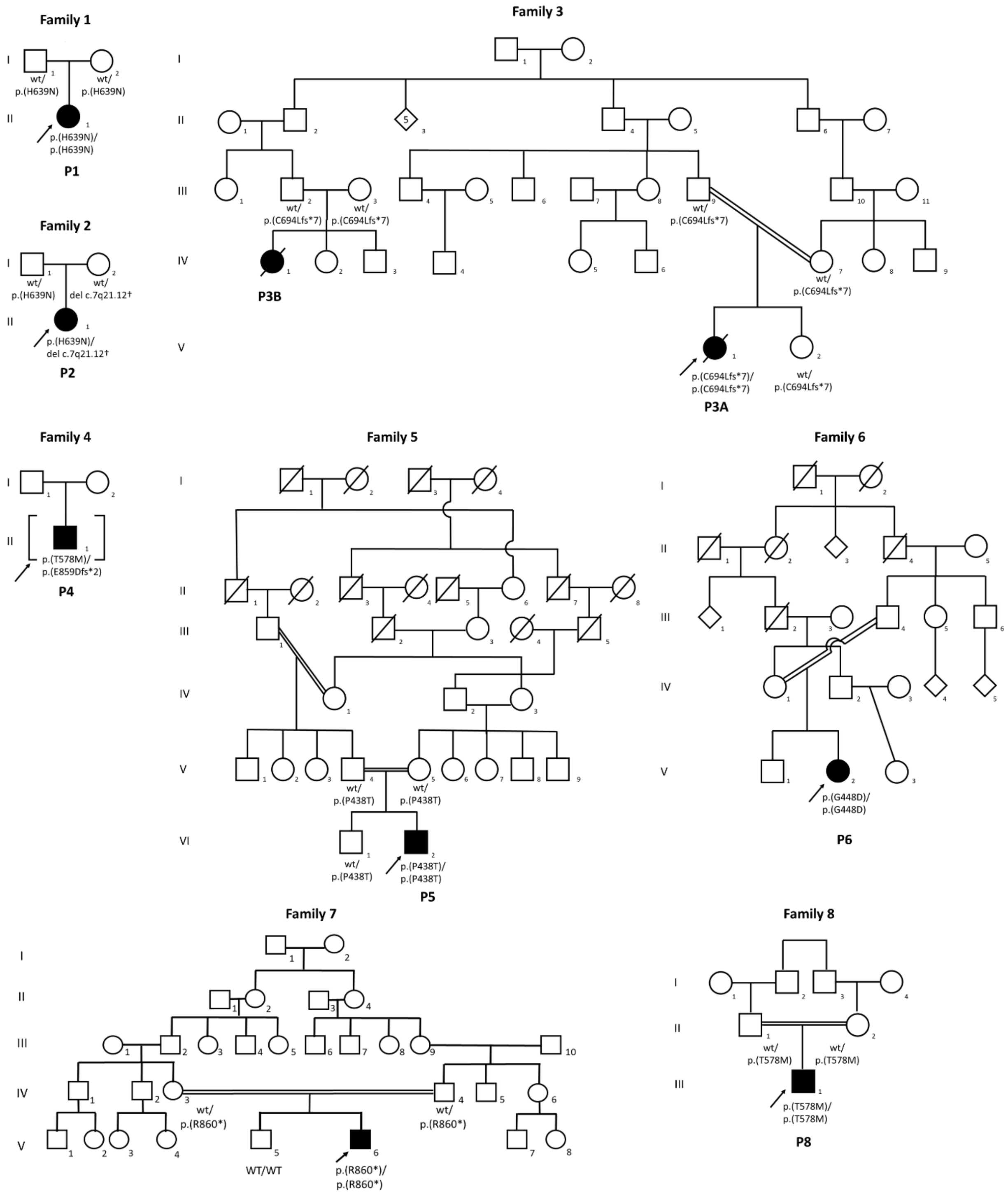

Supplementary Fig. 1 (continued)

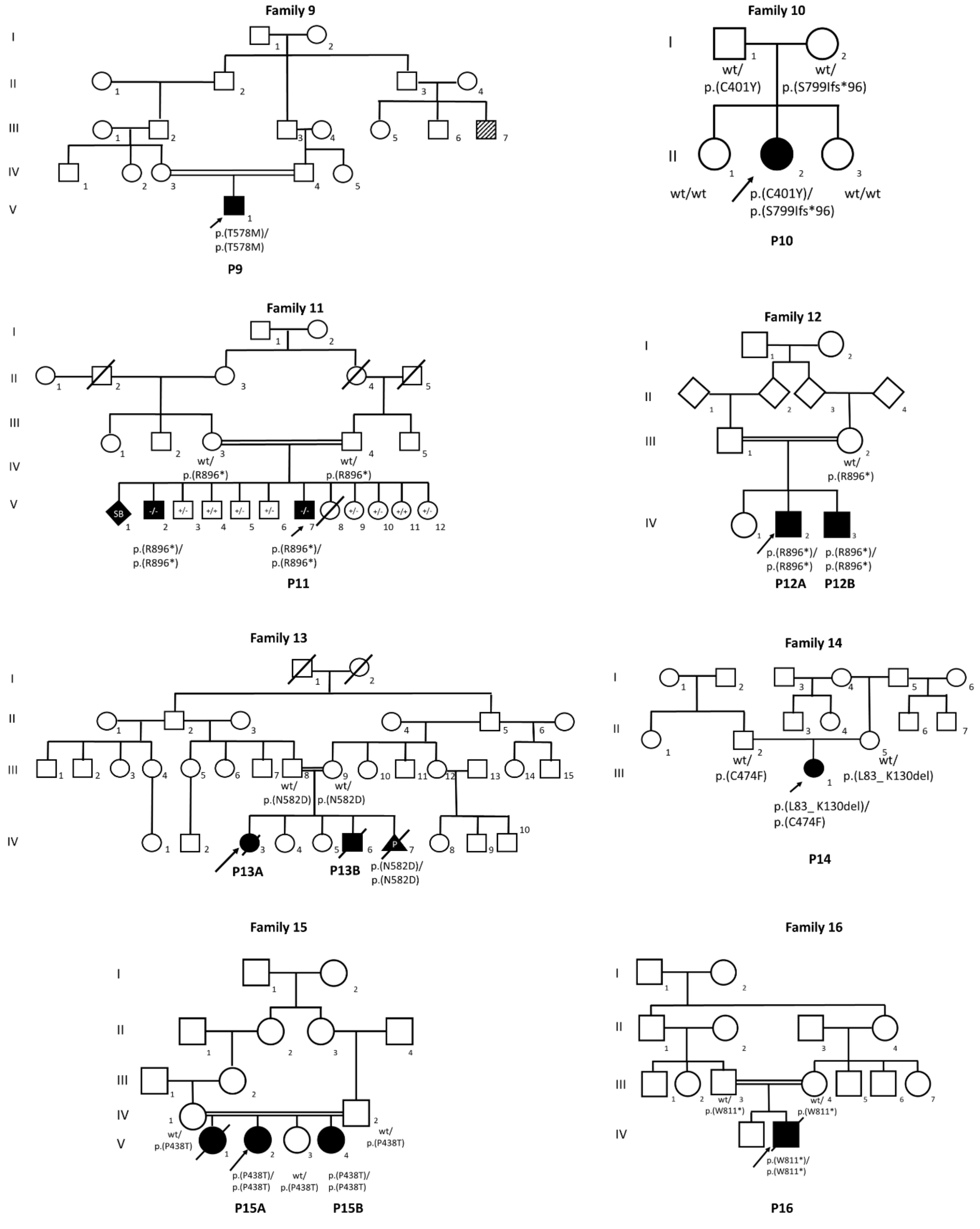

Supplementary Fig. 2

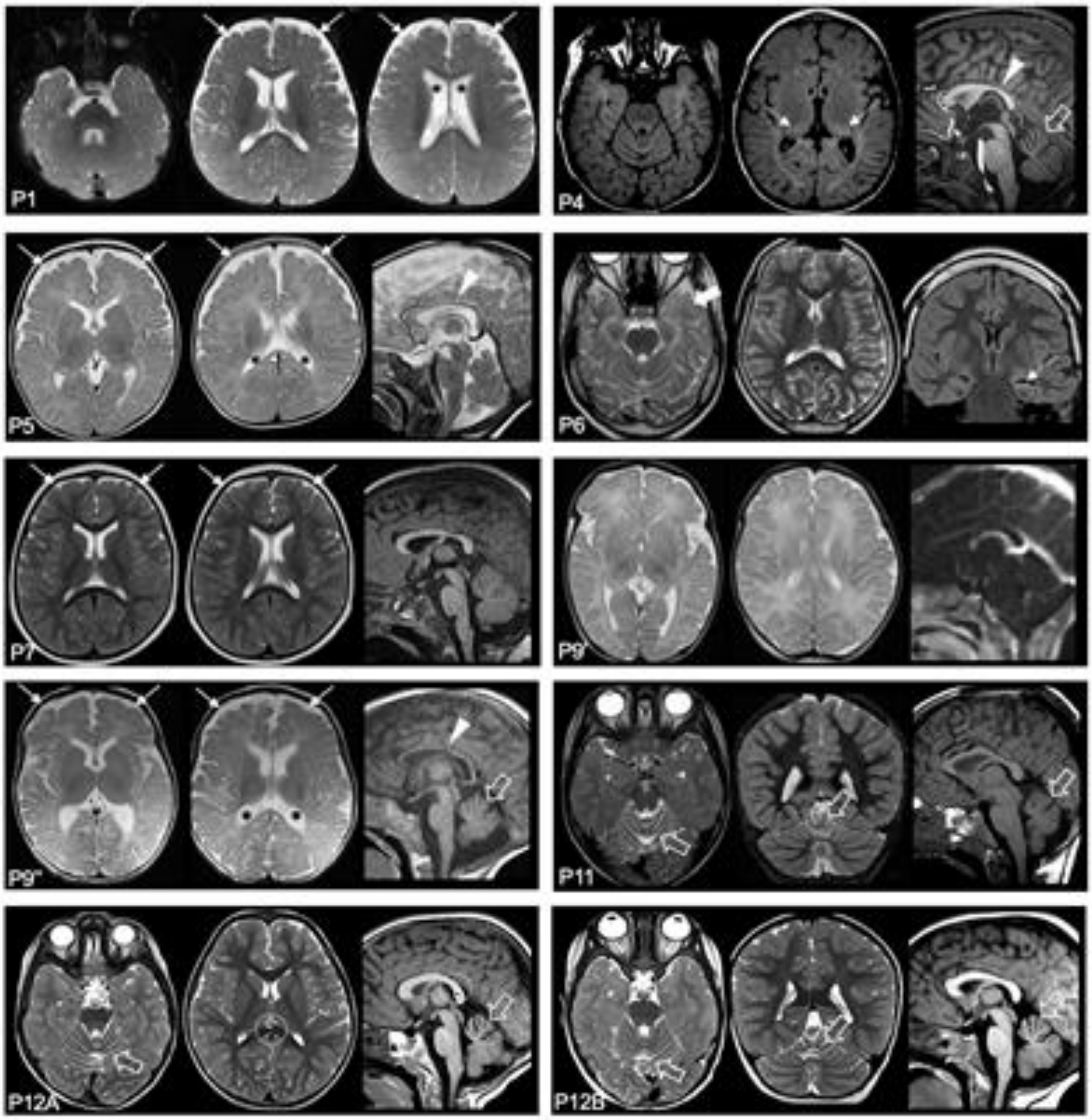

Supplementary Fig. 3

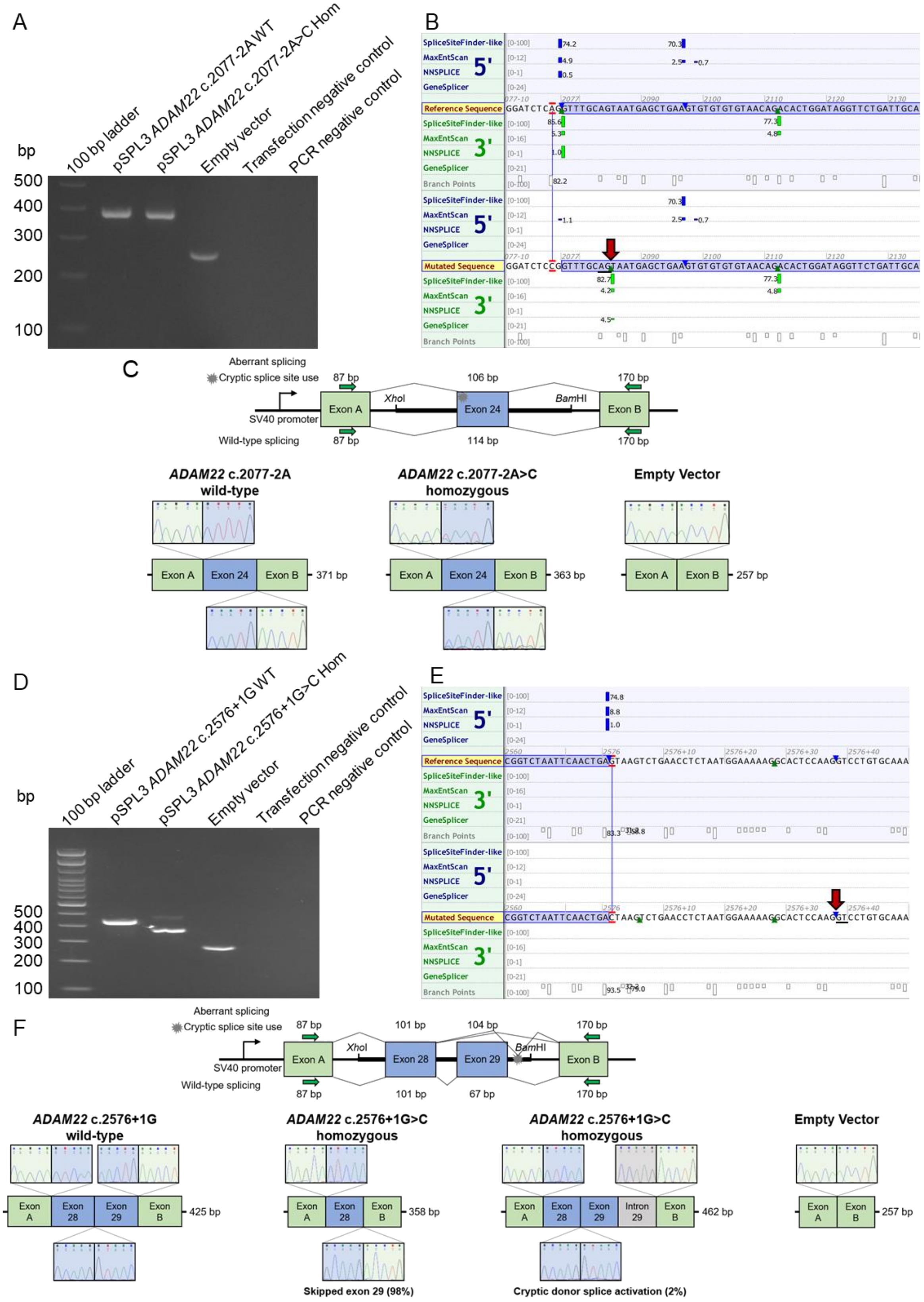

Supplementary Fig. 4

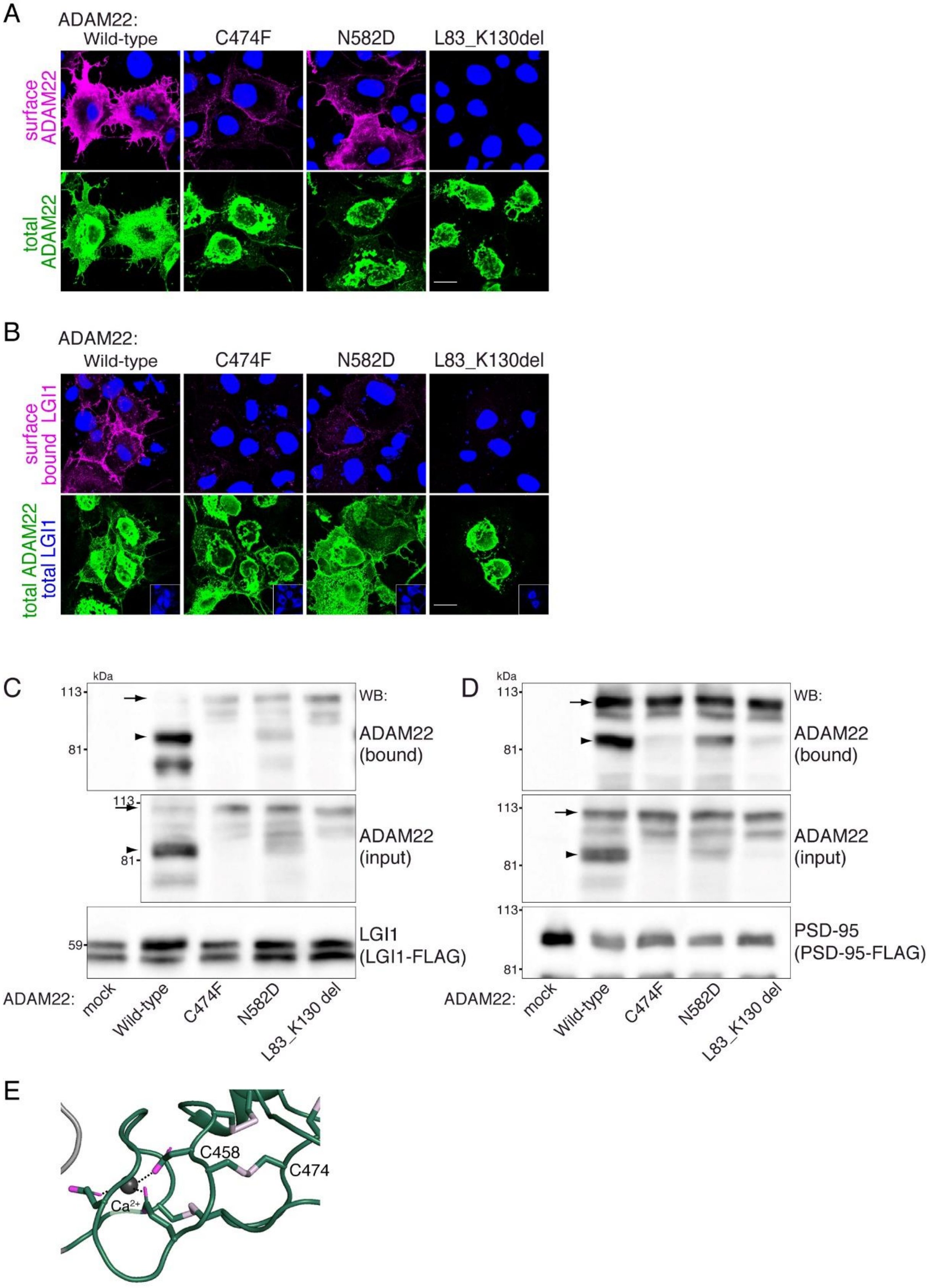

Supplementary Fig. 5

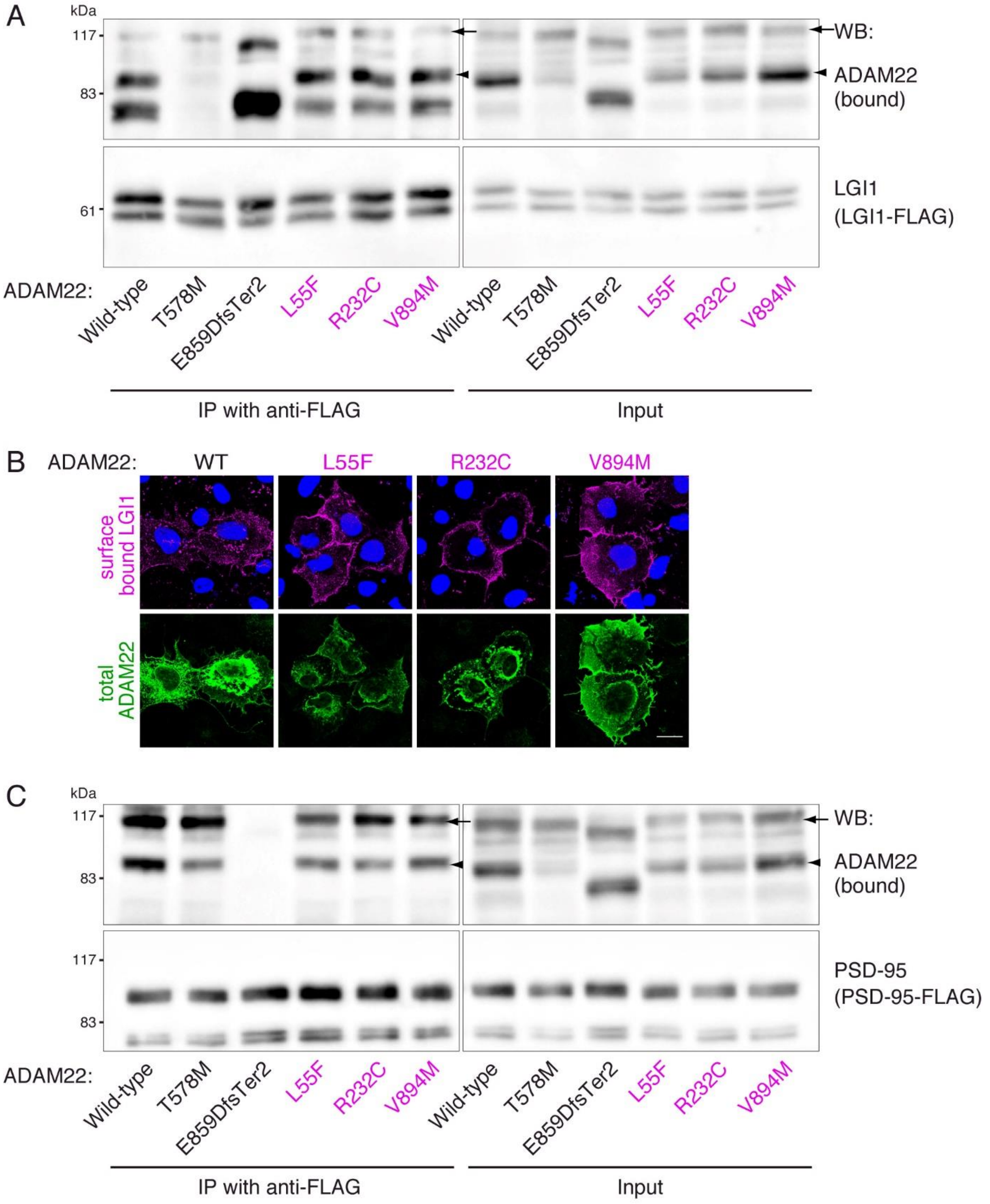

Supplementary Fig. 6

Related to Fig. 2B

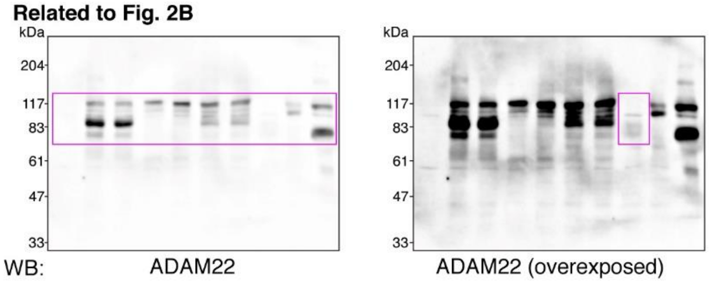

Related to Fig. 3A

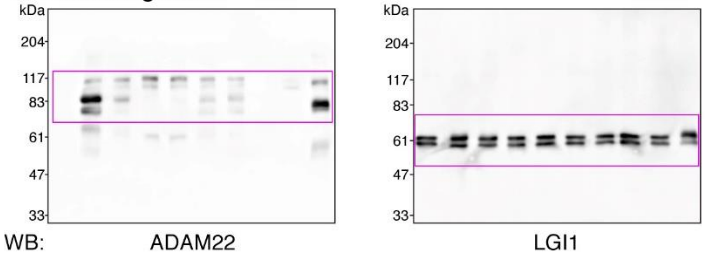

Related to Fig. 3C

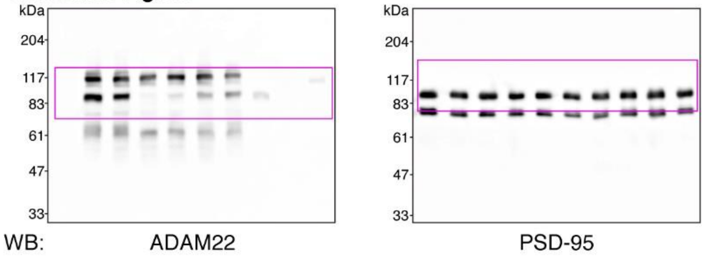

Related to supplementary Fig. 4C

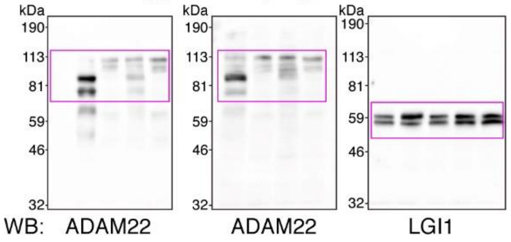

Related to supplementary Fig. 4D

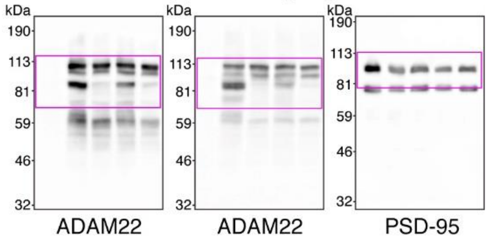

Related to supplementary Fig. 5A

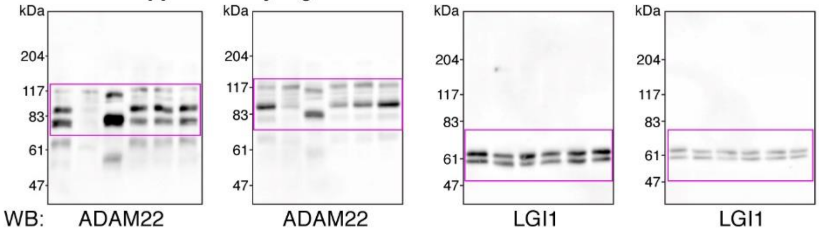

Related to supplementary Fig. 5C

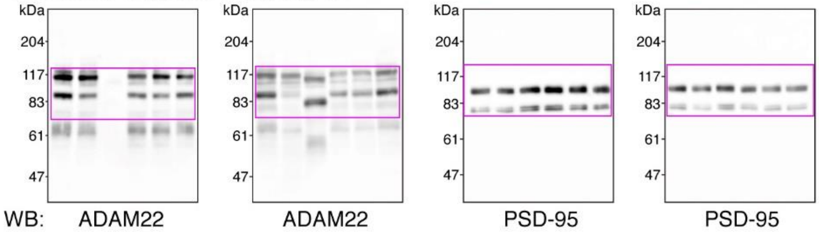

Supplement: awac116_Supplementary_Data [file awac116_supplementary_data.zip › brain-2021-02009-File006.pdf]
