# Supplementary material for: Biallelic ADAM22 pathogenic variants cause progressive encephalopathy and infantile-onset refractory epilepsy
Source: Brain. 2022 Apr 4;145(7):2301–12. doi: 10.1093/brain/awac116 (PMC9337806; doi:10.1093/brain/awac116)
Supplement: awac116_Supplementary_Data [file awac116_supplementary_data.zip › brain-2021-02009-File007.pdf]

Supplementary data. EEG findings in patients with biallelic ADAM22 mutations.

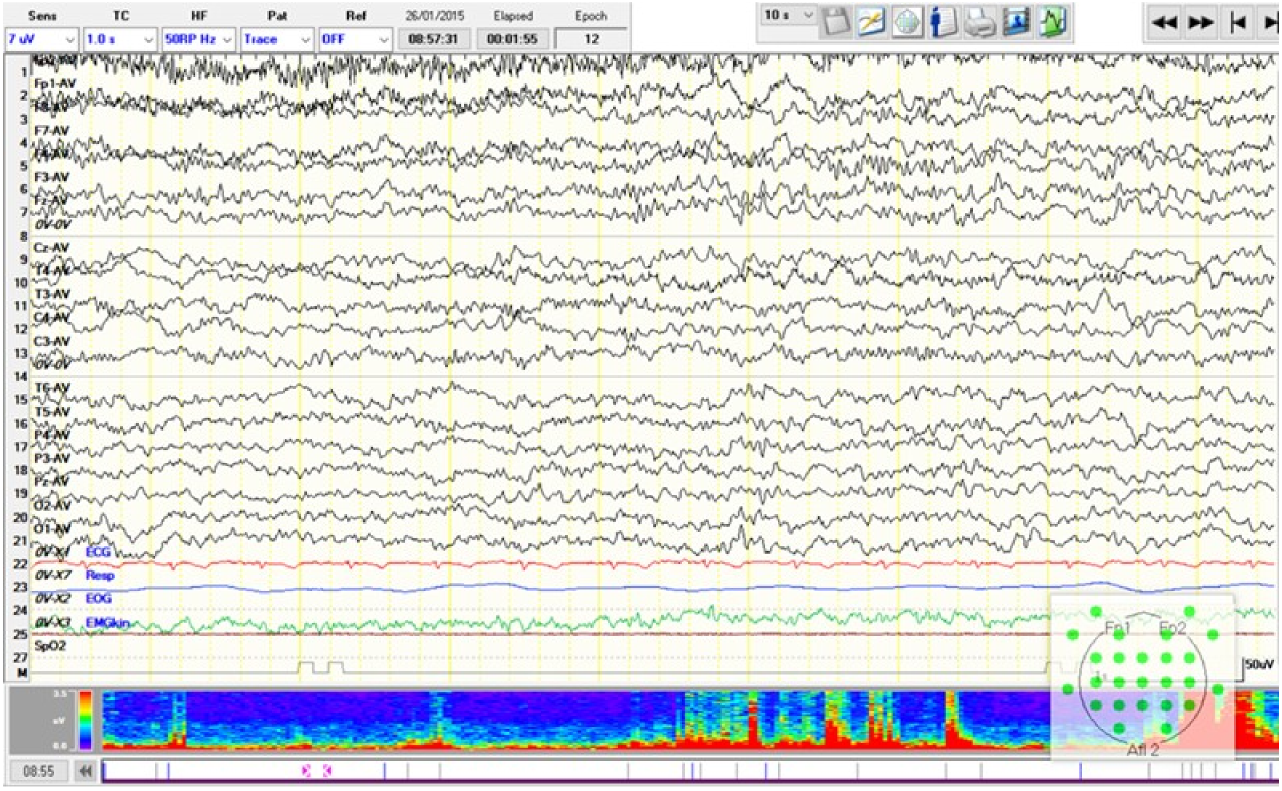

Interictal EEG from P1 at age 5 years showing diffusely low-voltage background activity and rare, small sharp waves on the central areas.

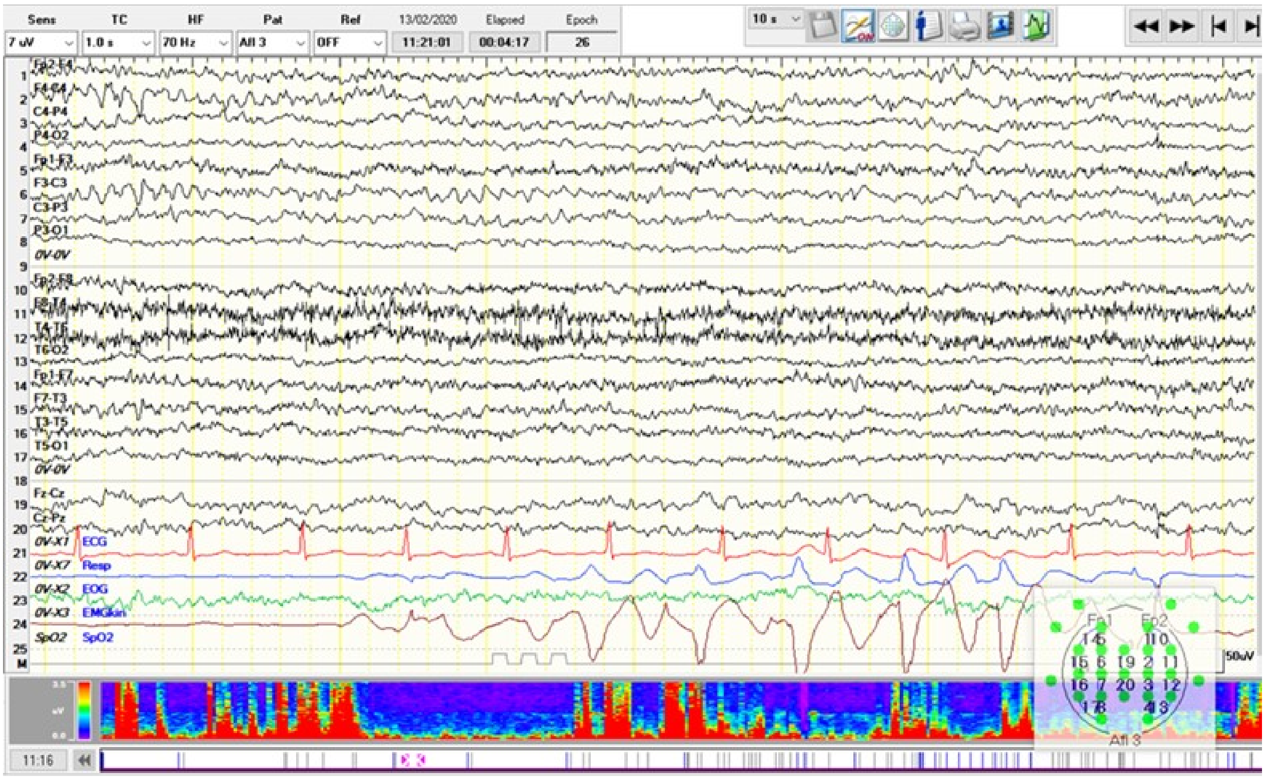

Interictal EEG from P1 at age 10 years showing diffuse slowed and dysregulated background activity and occasional theta rhythms over the frontal and central areas of both hemispheres.

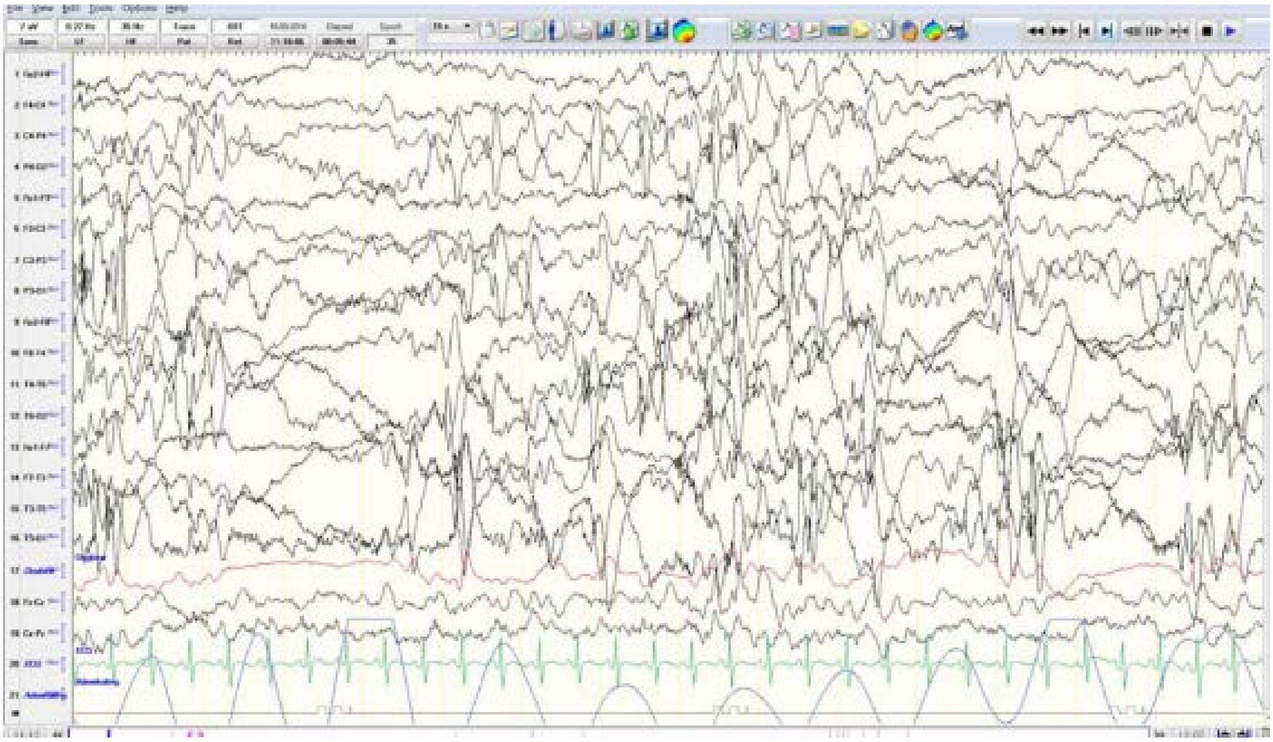

Interictal EEG from P2 at age 9 months, showing frequent multifocal epileptiformic abnormalities at the left more than the right side, increasing during sleep.

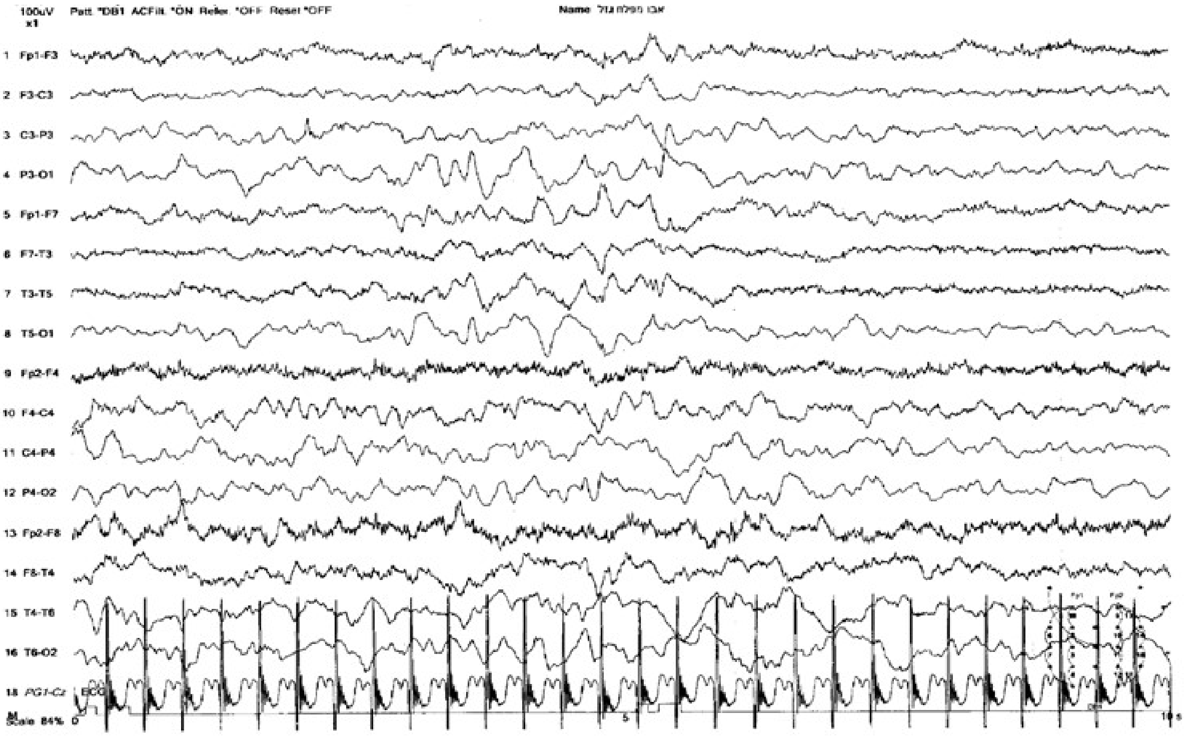

Interictal EEG from P3A at age 3 months showing irregular, slow wave and sharp wave complexes over the posterior left regions within a diffusely disorganized and slowed background activity.

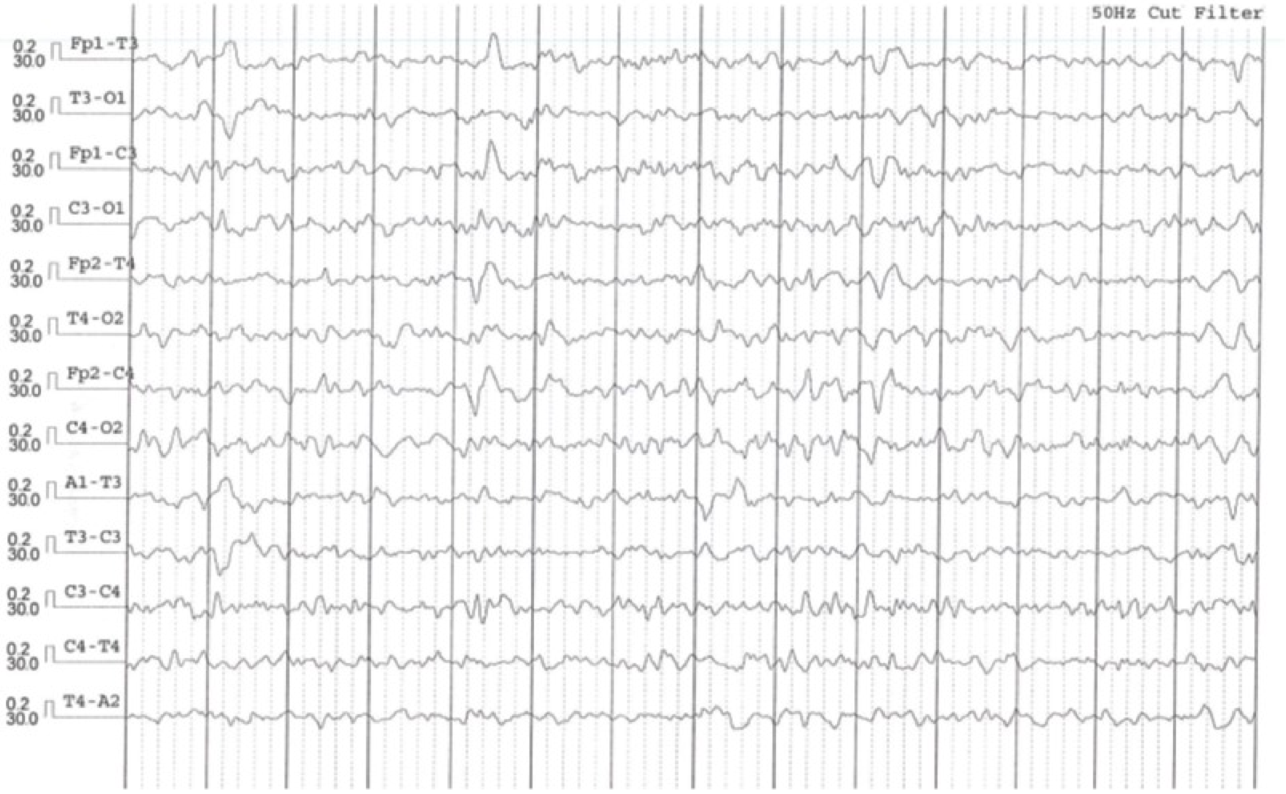

Interictal EEG from P5 (age 12 days) showing multifocal sharp waves and theta activities prominent over the central and temporal bilateral areas with disorganized background activity.

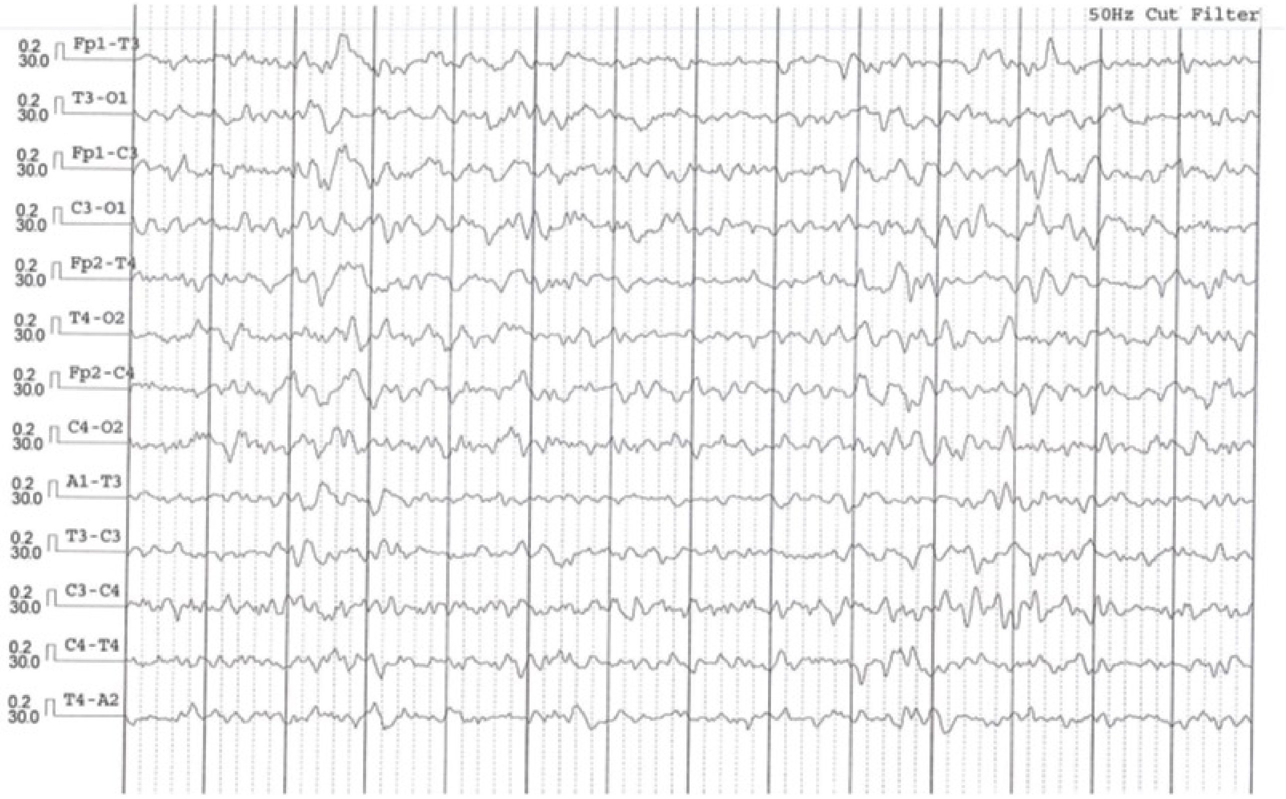

Interictal EEG from P5 (age 1 month) shows increased multifocal paroxysmal activity and predominance of theta activities over the central and posterior regions of both hemispheres.

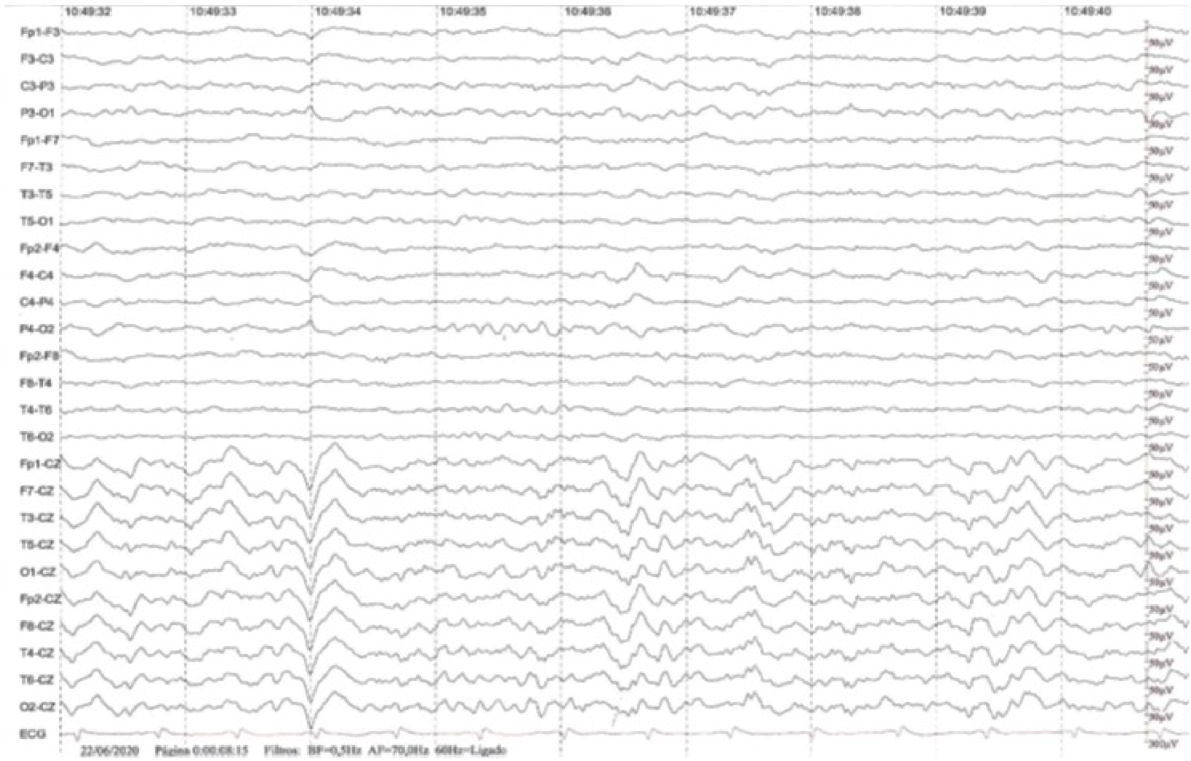

Interictal EEG from P6 at age 18 years showing diffuse slowed and dysregulated background activity and occasional slowing over the (L>R) central and temporal regions.

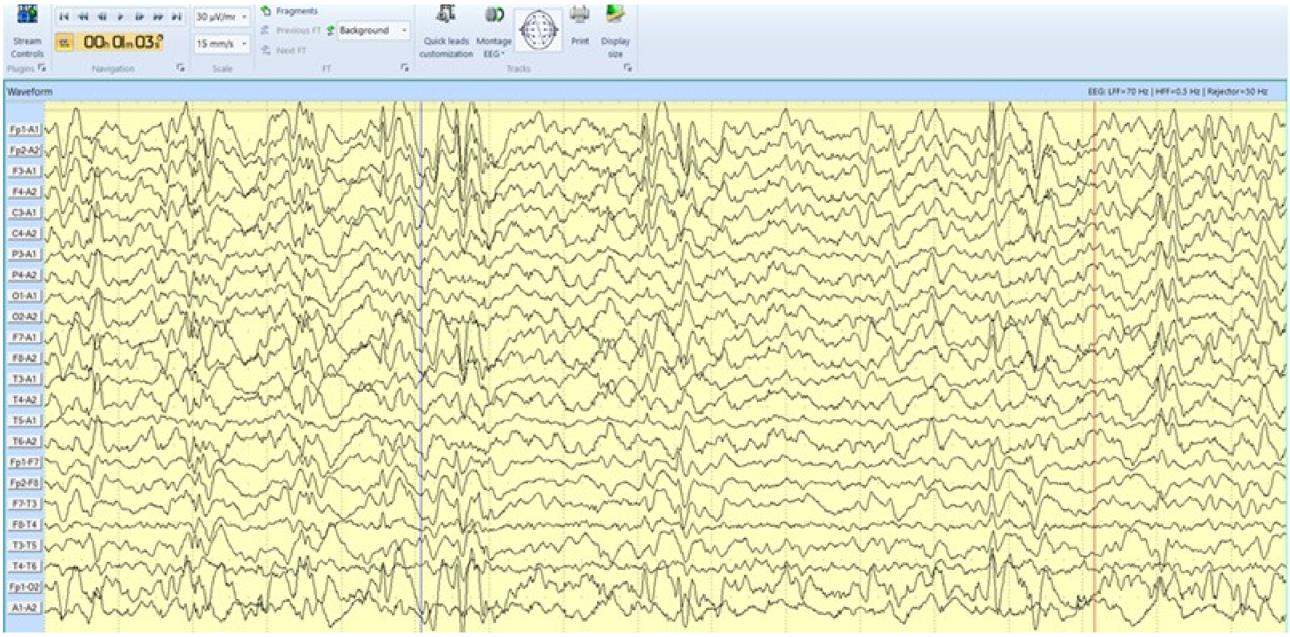

Interictal EEG from P7 showing high-amplitude and irregular, multifocal (L>R) waves and spikes/sharp waves within a background of chaotic and disorganized activity (*hypsarrhythmia).*

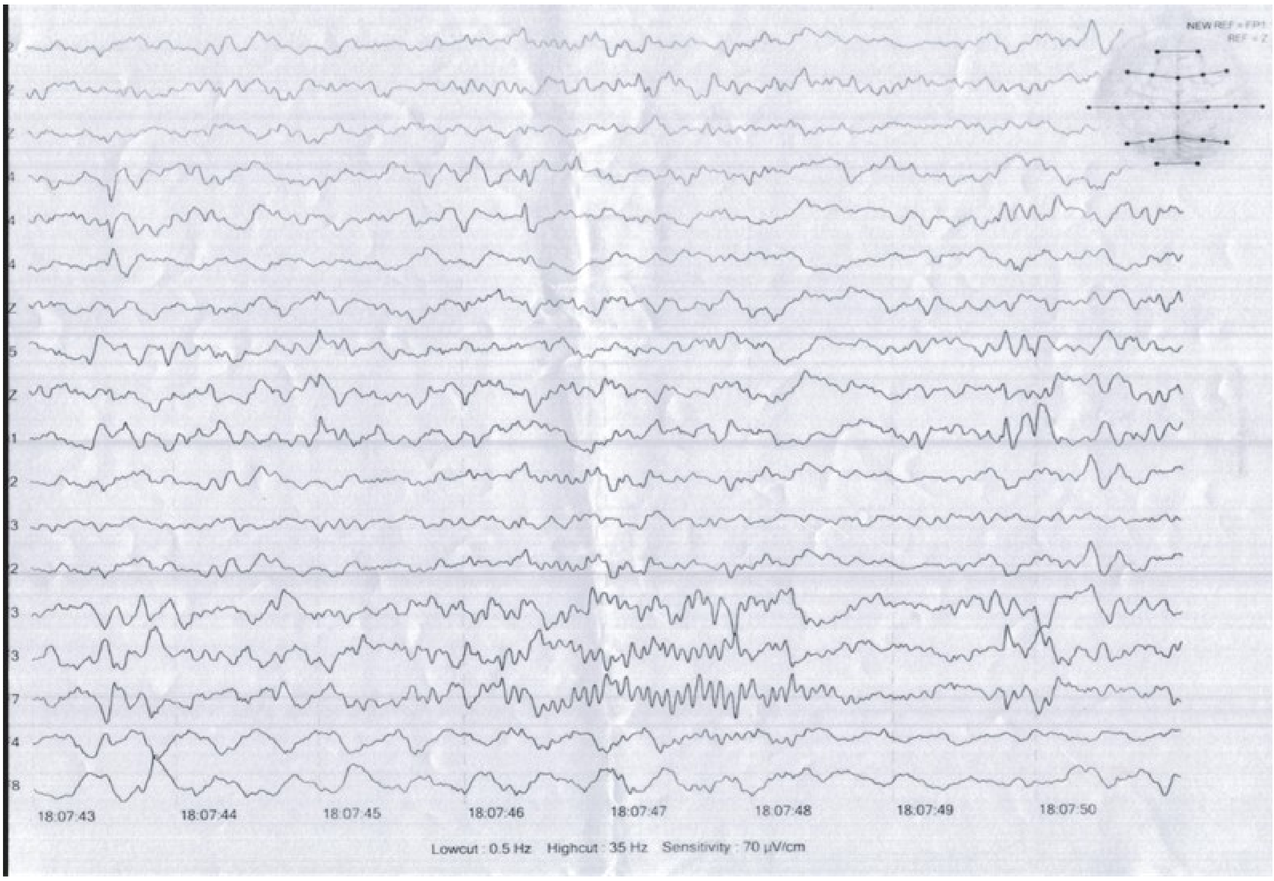

Interictal EEG from P9 at age 5 months showing irregular, multifocal sharp waves prominent over the central regions within a diffusely disorganized and slowed background activity.
